# High glucose induced c-Met activation promotes aggressive phenotype and regulates expression of glucose metabolism genes in HCC cells

**DOI:** 10.1038/s41598-021-89765-5

**Published:** 2021-05-31

**Authors:** Hande Topel, Ezgi Bağırsakçı, Yeliz Yılmaz, Ayşim Güneş, Gülsün Bağcı, Dehan Çömez, Erkan Kahraman, Peyda Korhan, Neşe Atabey

**Affiliations:** 1 Izmir Biomedicine and Genome Center (IBG), Balcova, 35340 Izmir, Turkey; 2 Department of Medical Biology and Genetics, Graduate School of Health Sciences, Dokuz Eylul University, Balcova, 35340 Izmir, Turkey; 3 Department of Molecular Biology and Genetics, Izmir International Biomedicine and Genome Institute, Dokuz Eylul University, Balcova, 35340 Izmir, Turkey

**Keywords:** Cancer microenvironment, Hepatocellular carcinoma, Epithelial-mesenchymal transition, Cell signalling, Cancer, Cell biology

## Abstract

Hepatocellular carcinoma (HCC) is strongly associated with metabolic dysregulations/deregulations and hyperglycemia is a common metabolic disturbance in metabolic diseases. Hyperglycemia is defined to promote epithelial to mesenchymal transition (EMT) of cancer cells in various cancers but its molecular contribution to HCC progression and aggressiveness is relatively unclear. In this study, we analyzed the molecular mechanisms behind the hyperglycemia-induced EMT in HCC cell lines. Here, we report that high glucose promotes EMT through activating c-Met receptor tyrosine kinase via promoting its ligand-independent homodimerization. c-Met activation is critical for high glucose induced acquisition of mesenchymal phenotype, survival under high glucose stress and reprogramming of cellular metabolism by modulating glucose metabolism gene expression to promote aggressiveness in HCC cells. The crucial role of c-Met in high glucose induced EMT and aggressiveness may be the potential link between metabolic syndrome-related hepatocarcinogenesis and/or HCC progression. Considering c-Met inhibition in hyperglycemic patients would be an important complementary strategy for therapy that favors sensitization of HCC cells to therapeutics.

## Introduction

Hepatocellular carcinoma (HCC) constitutes the majority (75–90%) of primary liver cancers^1^. Described risk factors of HCC include hepatitis B virus (HBV) and hepatitis C virus (HCV) infection, exposure to dietary aflatoxin, fatty liver disease, alcohol induced cirrhosis, obesity, smoking, diabetes and iron overload. Even many preventive strategies such as hepatitis immunization, lifestyle modifications and population screening are developed to reduce incidence of HCC, prevalence of HCC is still increasing^2^. Liver cancer is reported as one of the metabolism associated cancers and increasing incidence of liver cancer is explained by contribution of metabolic dysregulations such as obesity and type 2 diabetes mellitus (T2DM)^3^.

Hyperglycemia, as a result of metabolic conditions such as insulin resistance, obesity and T2DM, contributes to progression of many cancer types^3–5^. Besides inducing proliferation, invasion and motility, high glucose can modulate various signaling pathways. Inadequate maintenance of blood glucose was reported to be significantly associated with recurrence and poor survival of HCC patients after therapy^6^. Additionally, hyperglycemia has been reported to induce EMT in many cancer cells through aberrant activation of various oncogenic pathways^7–9^.

c-Met, a membrane receptor tyrosine kinase, is upregulated in liver diseases and favors hepatocyte proliferation, plasticity and regeneration^10^. In addition to its potential benefits in chronic liver diseases, c-Met activation induces initiation, development and progression of HCC through inducing EMT and promoting aggressive phenotype^10,11^. c-Met is an important component of EMT and mesenchymal phenotype in HCC^12^.

Metabolic reprogramming is a crucial component of epithelial/mesenchymal (E/M) phenotype switch but also, cellular metabolism has been described as an upstream regulator of cellular plasticity^13^. Accumulating data suggest that molecular reprogramming of metabolism and EMT are interdependent events and, both potentially modulate the other^13^. Metabolic reprogramming considered as a hallmark of cancer^14^ and increasing evidence indicate that mesenchymal cancer cells have different metabolic needs compared to their epithelial counterparts to satisfy the metabolic demands of motility and invasion^15^.

However, the regulatory molecular network between EMT and metabolic reprogramming and contribution of c-Met signaling to this interaction is still relatively unclear.

In this study, we investigate the role of c-Met signaling in hyperglycemia-induced EMT and reprogramming of glucose metabolism in HCC cells.

## Results

### High glucose promotes mesenchymal phenotype and induces motility and invasion in HCC cell lines

**Figure 1:**
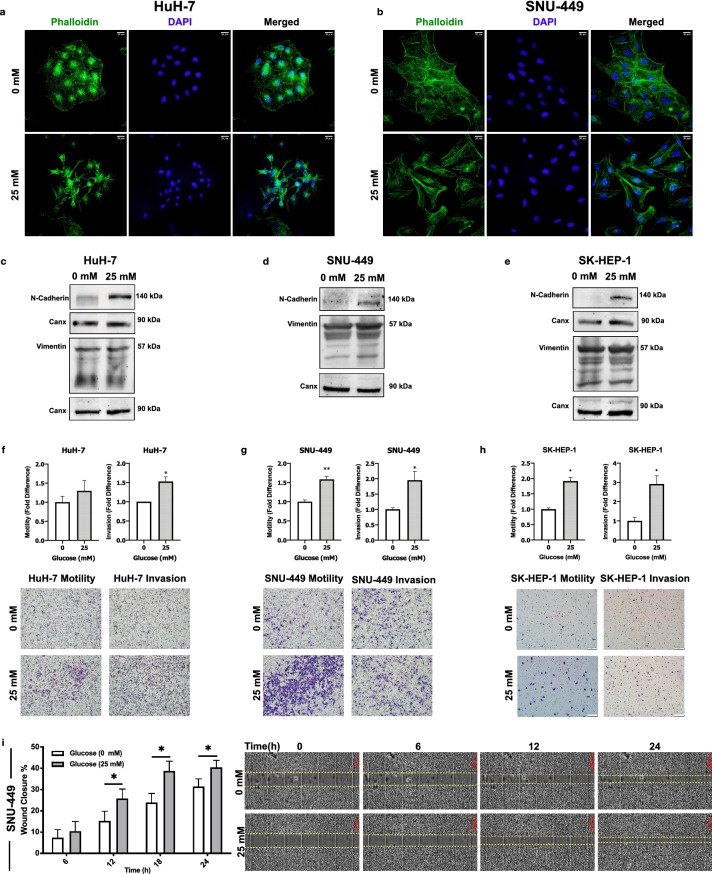
High glucose promoted mesenchymal phenotype and induced motility and invasion in HCC cell lines. Immunofluorescence imaging of Alexa-488-conjugated Phalloidin labeling of F-actin stress fibrils in (**a**) HuH-7 and (**b**) SNU-449 cells in no- and high-glucose (25 mM) supplemented culture conditions. Immunoblotting of cell cytoskeleton EMT biomarkers N-cadherin and Vimentin expressions in (**c**) HuH-7, (**d**) SNU-449 and (**e**) SK-HEP-1 cells. Graphical presentation of fold difference in motility and invasion of (**f**) HuH-7, (**g**) SNU-449 and (**h**) SK-HEP-1 cells cultured in no- and high-glucose supplemented media analyzed by trans-well motility and invasion assays. Graphical presentation of (**i**, left) wound closure percentage of SNU-449 cells after 6-, 12-, 18- and 24-h incubation in no- and high-glucose supplemented culture conditions. Wound-healing graphical presentation represents 3 independent experiments. Combined microscopic images of (**i**, right) SNU-449 wound healing assay from automated microscope for real-time live cell imaging on 0, 6th, 12th and 24th hours. Full length blots are presented in Supplementary. All graphs of experiments are presented as the mean ± SEM of at least 3 independent experiments. Statistical analyses were performed and column graphs were generated using GraphPad Prism version 8.2.1 for MacOS, GraphPad Software, San Diego, California USA, https://www.graphpad.com .* p ≤ 0.05, ** p ≤ 0.01, *** p ≤ 0.001, **** p ≤ 0.0001*.*

To investigate the effect of high glucose on E/M phenotype, we analyzed F-actin fibril organization in HuH-7 and SNU-449 cells via fluorescent-conjugated Phalloidin staining. High glucose exposure increased F-Actin staining intensity (Supplementary Fig. 1a) and modulated cellular morphology to attain spindle-like shape (Fig. 1a,b). To see the effect of high glucose on E/M molecular phenotype, we analyzed the expression of mesenchymal phenotype biomarkers N-Cadherin and Vimentin protein expression in HCC cells. High glucose induced N-Cadherin expression in all tested cell lines and Vimentin expression in SNU-449 (Fig. 1c–e).

Subsequently, we analyzed motility and invasion abilities of HuH-7, SNU-449 and SK-HEP-1 cells. High glucose exposure significantly increased motility and invasion capacity of SNU-449 and SK-HEP-1 whereas the increase in HuH-7 motility was not statistically significant (Fig. 1f–h). In addition to the increasing individual cell motility in trans-well assays, high glucose exposure enhanced wound closure ability of all cell lines tested (Fig. 1i, SNU-449; Supplementary Fig. 1b, HuH-7 and SK-HEP-1).

Briefly, high glucose induced acquisition of mesenchymal phenotype and enhanced motility and invasion ability of HCC cells.

### High glucose does not induce glucose toxicity but enhances spheroid formation in HCC cells

**Figure 2:**
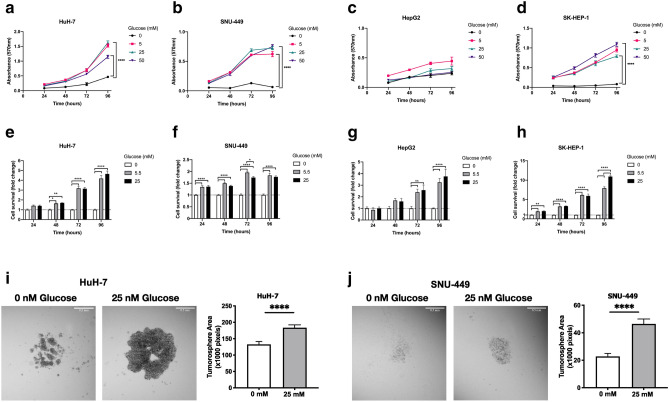
High glucose did not induce glucose toxicity but enhanced spheroid formation in HCC cells. Analysis of toxicity of (**a**) HuH-7, (**b**) SNU-449, (**c**) HepG2 and (**d**) SK-HEP-1 cells in response to increasing glucose concentrations (0, 5, 25, 50 mM) with MTT assay. Cell survival is represented as absorbance (570 nm). Analyses of cell survival differences of (**e**) HuH-7, (**f**) SNU-449, (**g**) HepG2 and (**h**) SK-HEP-1 cells in no-, normo- (5.5 mM) and hyperglycemic-glucose (25 mM) conditions by SRB. Brightfield images and graphical presentation of hanging-drop spheroid formation assay performed with (**i**) HuH-7 and (**j**) SNU-449 cells in no- and high-glucose (25 mM glucose) supplemented conditions. Spheroid area is calculated with ImageJ. All graphs of experiments are presented as the mean ± SEM of at least 3 independent experiments. Statistical analyses were performed and graphs were generated using GraphPad Prism version 8.2.1 for MacOS, GraphPad Software, San Diego, California USA, https://www.graphpad.com. * p ≤ 0.05, ** p ≤ 0.01, *** p ≤ 0.001, **** p ≤ 0.0001.

Increased glucose concentration in microenvironment promotes glucose-induced toxicity and suppresses cell viability in mammalian cells^16^. For culturing HCC cells which were used in this study, low glucose supplemented DMEM is recommended. To investigate whether glucose deprivation or high glucose concentrations promote cell death, we analyzed cell viability and proliferation with MTT assay under conditions with increasing glucose concentrations starting from 0 to 50 mM. MTT assay was performed with HuH-7, HepG2, SK-HEP-1 and SNU-449 cell lines which were cultured with glucose deprived (no-glucose, 0 mM) or normo-glucose (5.5 mM) and high glucose (25–50 mM) supplemented media. As expected, HCC cells were resistant to glucose toxicity and glucose treatment did not show any detrimental effect on cell viability rather induced proliferation in tested concentrations. Additionally, glucose deprivation did not induce cell death but lowered the proliferation rate (Fig. 2a–d). To investigate the effect of glucose level on survival of HCC cells, we performed sulforhodamine (SRB) assay with HuH-7, HepG2, SK-HEP-1 and SNU-449 cells. As expected, high glucose did not affect survival of HCC cell lines (Fig. 2e–h).

Spheroid formation ability and survival in adhesion-independent conditions are two important markers of aggressive cell behavior in cancer progression and metastasis^17^. To investigate their spheroid formation and adhesion-independent survival ability, HuH-7 and SNU-449 cells were cultured in hanging droplets of starvation and high glucose (25 mM) culture media. Consistent with the viability and proliferation data, both cell lines expanded the dimensions of spheroids in high glucose conditions (Fig. 2i,j).

To summarize, high glucose treatment does not promote glucose induced toxicity in HCC cells but improves cell proliferation and spheroid formation abilities. Additionally, glucose deprivation does not promote cell death but lowers proliferation rate of HCC cells.

### High glucose exposure activates c-Met and downstream signaling via promoting ligand-independent homodimerization

**Figure 3:**
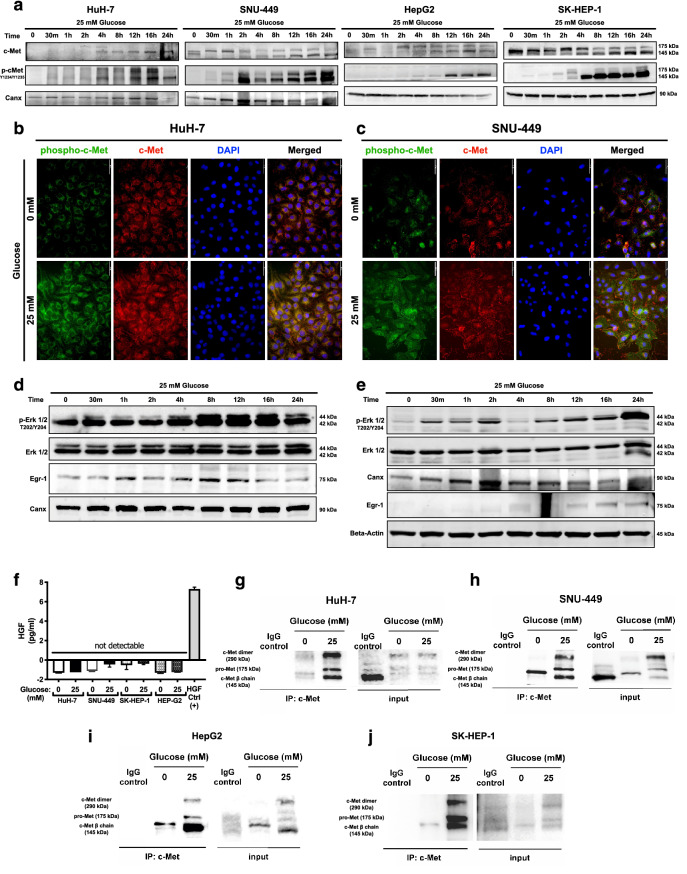
High glucose exposure activated c-Met and downstream signaling via promoting ligand-independent homodimerization. (**a**) Immunoblotting of time-dependent c-Met protein expression and activation phosphorylations (Y1234/Y1235) in response to high glucose treatment in HuH-7, HepG2, SNU-449, and SK-HEP-1 cells. Immunofluorescence imaging of Alexa-594-labeled c-Met protein and Alexa-488 labeled c-Met activation phosphorylations (Y1234/Y1235) in response to no- and high-glucose (25 mM) conditions in (**b**) HuH-7 and (**c**) SNU-499 cells. Cell nuclei were counterstained with DAPI staining. Immunoblotting of time-dependent expressions of Erk1/2 activation phosphorylations (T202/Y204), Erk1/2 total protein and Egr-1 protein in response to high glucose stimulation in (**d**) HuH-7 and (**e**) SNU-449 cells. Calnexin (Canx) immunoblotting performed as an internal control. (**f**) Graphical presentation of HGF secretion analysis with ELISA. Conditioned media of HCC cells cultured in no- and high-glucose supplemented conditions were analyzed with sandwich ELISA. Crosslinking-immunoprecipitation assay for analyzing c-Met homodimerization under no- and high-glucose (25 mM) conditions in (**g**) HuH-7, (**h**) SNU-449, (**i**) HepG2 and (**j**) SK-HEP-1 cells. After starvation and indicated treatments for 16 h, proteins were crosslinked with Sulfo-EGS, c-Met was immunoprecipitated and immunoblotted with c-Met targeting primary antibody. Full length blots are presented in Supplementary. Column graph was generated using GraphPad Prism version 8.2.1 for MacOS, GraphPad Software, San Diego, California USA, https://www.graphpad.com.

Induction of mesenchymal phenotype and aggressive behavior are well-defined consequences of c-Met activation in HCC^18–20^. To understand the contribution of c-Met signaling to high glucose induced EMT, we investigated c-Met expression and activation in response to high glucose exposure. Time-dependent activation and expression of c-Met receptor tyrosine kinase were analyzed in HuH-7, HepG2, SNU-449 and SK-HEP-1 cell lines and high glucose exposure increased c-Met activation (phospho-Met Y1234/Y1235), protein expression (Fig. 3a) and transcription (Supplementary Fig. 1c) in all HCC cell lines tested. Induction of c-Met activation and expression were confirmed with immunofluorescence staining of c-Met activatory phosphorylations and total c-Met protein in HuH-7 and SNU-449 cells (Fig. 3b,c). When compared with the physiological glucose levels (5.5 mM), high glucose increases c-Met activation (phospho-Met, Y1234/Y1235) and expression in HCC cell lines (Supplementary Fig. 1d). To investigate whether glucose-induced c-Met activation promotes signal transduction through downstream, secondary effectors of c-Met signaling were analyzed by immunoblotting. We analyzed activation (phospho-Erk1/2, T202/Y204) and expression of Erk1/2 and expression of Egr-1, a well-defined EMT-related transcription factor^21^, in HuH-7 and SNU-449 cells. High glucose treatment increased Erk1/2 activation and Egr-1 expression in SNU-449 and HuH-7 cells (Fig. 3d,e).

Activation mechanisms of c-Met receptor tyrosine kinase vary according to the cellular contexts and microenvironment components^11^. To test the potential role of autocrine HGF secretion in high glucose induced c-Met activity, we analyzed HGF concentrations in conditioned media of HCC cells via sandwich ELISA method. HGF levels were not detectable in both starvation and high glucose conditions, which indicate high glucose induces c-Met activation in a ligand-independent manner (Fig. 3f).

For acquiring a better understanding of glucose-induced c-Met activation mechanism, we analyzed c-Met homodimerization in response to high glucose exposure by immunoprecipitating c-Met with prior use of the crosslinker Sulfo-EGS in HCC cells. High glucose exposure promoted homodimerization of c-Met in HuH-7, SNU-449, HepG2 and SK-HEP-1 cell lines (Fig. 3g–j).

To summarize, high glucose exposure induces expression and activation through receptor homodimerization and downstream signal transduction of c-Met but does not promote autocrine HGF secretion in HCC cells.

### c-Met activity is crucial for acquisition of high glucose induced aggressive behavior

**Figure 4:**
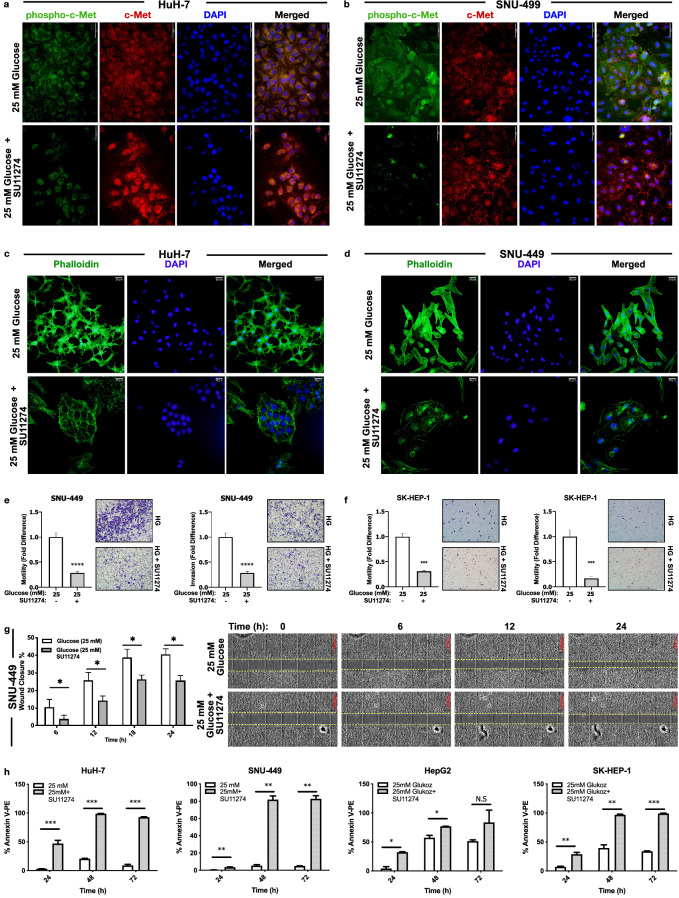
High glucose increased cell aggressiveness reversed by inhibition of c-Met activation. Representative images showing protein expression of c-Met (Alexa-594 labeled) and phospho-Met (Y1234/Y1235), (Alexa-488 labelled) in (**a**) HuH-7 and (**b**) SNU-449 cells. Representative images showing F-actin stress fibrils (Alexa-488-conjugated Phalloidin labelled) in (**c**) HuH-7 and (**d**) SNU-449 cells. Cell nucleus is counterstained with DAPI staining. Graphical presentation of fold change of (**e**) motility and invasion in SNU-449 and (**f**) SK-HEP-1 cells in trans-well motility and invasion assays. Graphical presentation of (**g**) wound closure percentage of SNU-449 cells after 6-, 12-, 18- and 24-h incubation. Wound-healing graphical presentation represents 3 independent experiments. Combined microscopic images of SNU-449 wound healing assay from automated microscope for real-time live cell imaging on 0, 6th, 12th and 24th hours. Percentage of Annexin-V/PI positive HCC cells (**h**) in high glucose and c-Met inhibition conditions measured by flow cytometry. All data represented in this figure are from indicated HCC cells that are cultured with high-glucose control and high-glucose + SU11274 (2.5 µM) supplemented conditions. All graphs of experiments are presented as the mean ± SEM of at least 3 independent experiments. Statistical analyses were performed and graphs were generated using GraphPad Prism version 8.2.1 for MacOS, GraphPad Software, San Diego, California USA, https://www.graphpad.com. *p ≤ 0.05, ** p ≤ 0.01, *** p ≤ 0.001, **** p ≤ 0.0001*.*

To test the necessity of c-Met activation in acquisition of high glucose induced aggressive phenotype in HCC, we performed a set of experiments by inhibiting c-Met activity with a c-Met specific small molecule inhibitor, SU11274. c-Met inhibitor was effective to repress high-glucose induced c-Met activation in HuH-7, SNU-449, HepG2 and SK-HEP-1 cells (Supplementary Fig. 2a). Additionally, we analyzed and visualized the inhibition of c-Met activity by SU11274 via immunofluorescence staining of c-Met activation (phospho-Met, Y1234/Y1235) and total protein expression in HuH-7 (Fig. 4a) and SNU-449 (Fig. 4b) cells.

After confirmation of efficient inhibition of c-Met activity, we investigated the effects of c-Met inhibition on high glucose induced EMT and mesenchymal phenotype. Specific inhibition of c-Met kinase activity changed cell morphology from spindle-shape (mesenchymal-like) to wide-round (epithelial-like) cell shape (Fig. 4c,d) and decreased F-actin fibril staining intensity (Supplementary Fig. 1a) in HuH-7 and SNU-449 cells. To investigate the effect of c-Met inhibition on aggressiveness, we analyzed wound healing, motility and invasion capacity of HCC cells. c-Met activity inhibition significantly repressed trans-well migration and invasion capacity of HCC cells (Fig. 4e,f). Wound closure capacity of cells was decreased in all tested HCC cell lines (Fig. 4g, SNU-449; Supplementary Fig. 1b, HuH-7 and SK-HEP-1).

As reported in previous studies, high glucose induces apoptosis in normal mammalian cells whereas resistance to apoptosis is defined as a distinctive cancer hallmark^14,22,23^. To address the effect of c-Met kinase activity inhibition on resistance to glucose toxicity, we analyzed apoptotic activity of HCC cells with Annexin-V/PI staining in flow cytometry. Inhibition of c-Met kinase activity increased sensitivity to glucose toxicity by promoting apoptosis in all HCC cell lines tested (Fig. 4h).

Overall, c-Met is required to acquire high glucose induced aggressive phenotype in HCC cells. High glucose induced aggressive phenotype can be reversed by c-Met inhibition.

### Inhibition of c-Met kinase activity reverses glycolytic gene expression pattern in HCC cells

Having observed the significant regulatory role of high glucose on c-Met signaling pathway in-vitro, we performed bioinformatic analysis to see the effect of c-Met expression level in patients’ data. First, we evaluated the effect of MET expression on cellular pathways and gene transcription profiles. We analyzed TCGA HCC tissue sample gene expression data by grouping the tumor tissue samples into quartiles according to their normalized MET mRNA expression levels. Then, we performed differential gene expression (DEG) analysis between low-*MET* expressing quartile (Q1) and high-*MET* expression quartile (Q4) and, analyzed enriched pathways associated with DEGs with ClusterProfiler^24^. In pathway enrichment analysis, carbohydrate metabolism was listed as one of the most over-represented pathways and in addition to carbohydrate metabolism, many metabolism pathways were enriched in pathway over-representation analysis (Supplementary Fig. 2b). DisGeNET^25^, gene-disease association enrichment analysis showed that Diabetes Mellitus and Hyperglycemia were in the list of the most associated diseases with the enriched DEGs (Supplementary Fig. 2c). After observing a strong relation with glucose metabolism in enrichment analyses, we analyzed and visualized fold change differences of DEGs in “central carbon metabolism in cancer” pathway with Pathview^26^. The expression of glucokinase, glucose transporter and glutaminase genes were significantly increased in high-*MET* expressing groups, whereas hexokinase, pyruvate kinase and phosphofructokinase genes were significantly decreased (Supplementary Fig. 2d).

**Figure 5:**
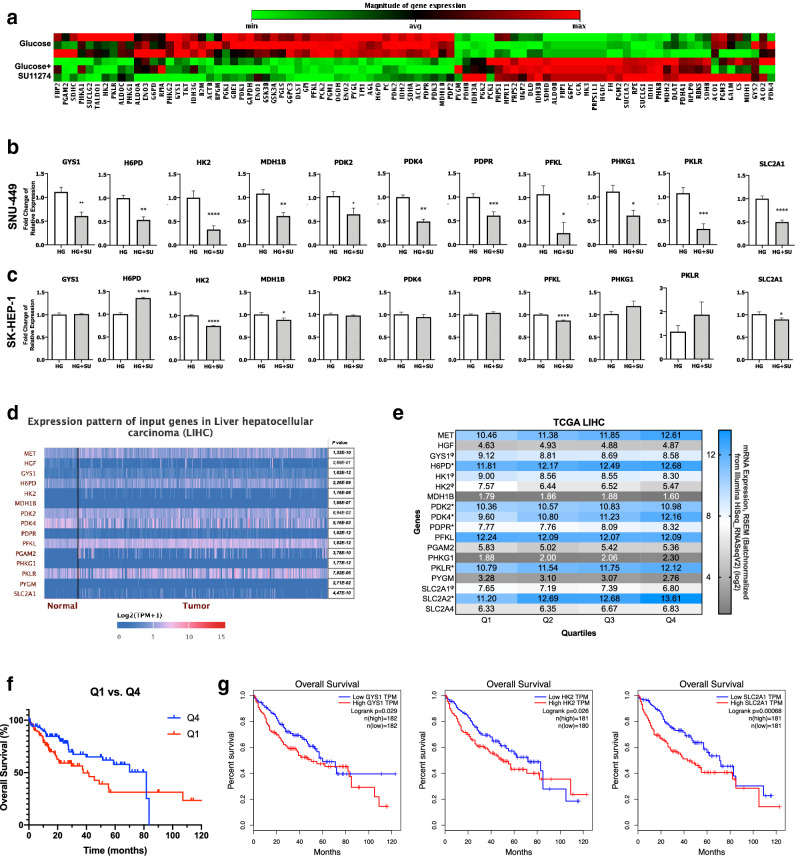
The effect of c-Met kinase activity inhibition on glycolytic gene expression patterns in HCC. Heat-map of glucose metabolism gene expression analysis by RT-qPCR array in SNU-449 cells cultured in high glucose (25 mM) and high glucose together with c-Met-specific kinase inhibitor SU11274 (2.5 μM) **(a)**. RT-qPCR validation of genes differentially expressed in RT-qPCR array experiments (GYS1, H6PD, HK2, MDH1B, PDK2, PDK4, PDPR, PFKL, PHKG1, PKLR) and glucose transporter GLUT1 (SLC2A1) in SNU-449 (**b**) and SK-HEP-1 cells treated with high glucose (25 mM) and high glucose together with c-Met-specific kinase inhibitor SU11274 (2.5 μM) **(c)**. Heat-map presentation of indicated genes’ expression in normal and tumor tissue samples from patients in TCGA-LIHC dataset. Expression heat-map and adjusted *p-value*s were generated with UALCAN from TCGA-LIHC dataset (**d)**. Comparative expression analysis of indicated genes in TCGA-LIHC quartiles that were grouped according to *MET* expression **(e)**. φ: significantly lower in high-*MET* quartile, *: significantly higher in high-*MET* quartile. Overall survival of patients in Low*-MET* and High*-MET* expression group based on TCGA data (**f**). Overall survival of patients in Low*-GYS1* and High*-GYS1* (left graph), Low*-HK2* and High*-HK2* (middle graph) and Low*-SLC2A1* and High*-SLC2A1* (right graph) expression groups based on TCGA data (**g**). Graphs (**g**) were generated by UALCAN. RT-qPCR graphs are presented as the mean ± SEM of at least 3 independent experiments. Statistical analyses were performed, and column graphs, heat-map image **(e)** and survival graph **(f)** were generated using GraphPad Prism version 8.2.1 for MacOS, GraphPad Software, San Diego, California USA, https://www.graphpad.com. * p ≤ 0.05, ** p ≤ 0.01, *** p ≤ 0.001, **** p ≤ 0.0001*.*

Having determined the potential role of c-Met on tumor metabolism by bioinformatic analysis, we tested the effects of c-Met inhibition on glucose metabolism genes. We analyzed expression of glucose metabolism genes by quantitative RT-PCR array in the presence of high glucose (25 mM) and/or c-Met inhibitor SU11274 in SNU-449 HCC cell line. The RT-PCR array was composed of the genes that function in glycolysis, glucose metabolism regulation, the citric acid cycle (TCA), pentose phosphate pathway (PPP) and glycogenesis. Heat-map analysis of glucose metabolism gene expression array revealed a significant switch in glucose metabolism related gene expression in response to inhibition of c-Met kinase activity (Fig. 5a). Differentially expressed gene (DEG) analysis revealed that inhibition of c-Met kinase activity repressed expression of genes taking part in glycolysis (*HK2, PFKL, PGAM2, PKLR*), glucose metabolism regulation (*PDK2, PDPR*), TCA cycle (*MDH1B*), pentose phosphate pathway (*H6PD*), glycogen synthesis (*GYS1*) and regulation (*PHKG1*) while upregulating *PYGM* gene that takes role in glycogen break-down, significantly. Subsequent quantitative RT-qPCR analysis was performed to confirm RT-PCR array results. In addition to the genes analyzed by RT-PCR array, we analyzed expression of glucose transporters (*GLUT*s) and pyruvate dehydrogenase kinases (*PDK*s). c-Met inhibition changed glucose metabolism related gene expression enormously in SNU-449 cells. Expression of *GYS1*, *H6PD*, *HK2*, *MDH1B*, *PDK2*, *PDK4*, *PDPR*, *PFKL*, *PHKG1*, *PKLR* and glucose transporter *SLC2A1 (GLUT1)* were repressed by inhibition of c-Met kinase activity in SNU-449 cells, significantly (Fig. 5b). In addition to SNU-449, we analyzed the expression of DEGs in SK-HEP-1 cell line but the effect of c-Met kinase activity inhibition on DEGs were different in SK-HEP-1 cells. c-Met tyrosine kinase inhibition repressed expression of *HK2*, *MDH1B*, *PFKL* and *SLC2A1*, whereas increased *H6PD* expression in SK-HEP-1 cells, significantly (Fig. 5c). Finally, we investigated the effects of high glucose induction and inhibition of c-Met kinase activity on oxygen consumption rate (OCR) and extracellular acidification rate (ECAR) in Met-expressing mouse-derived hepatoma cell line Hepa1-6 via Seahorse XFe96 instrument. Hepa1-6 cell line is a well-known mouse cell line with elevated expression and activation of c-Met^27^. First, we analyzed protein expression and activation of c-Met in low glucose (LG), high glucose (HG) and c-Met tyrosine kinase activity inhibitor supplemented high glucose conditions (HGi) to confirm the effect of high glucose exposure on c-Met activity and inhibitor efficiency (Supplementary Fig. 2e). High glucose exposure decreased OCR and did not change ECAR level when compared to normo-glucose condition. c-Met activity inhibition (HGi) improved OCR and decreased ECAR when compared to HG (Supplementary Fig. 2f).

Subsequently, we tested our in vitro findings by performing further bioinformatic analysis. We analyzed expression of *MET*, its ligand *HGF* and DEGs (*GYS1*, *H6PD*, *HK2*, *MDH1B*, *PDK2*, *PDK4*, *PDPR*, *PFKL*, *PHKG1*, *PKLR* and *SLC2A1*) in normal and tumor tissues of liver hepatocellular carcinoma (LIHC) cohort of The Cancer Genome Atlas (TCGA) via UALCAN^28^. Consistent with the literature, *MET* expression was significantly elevated in tumor tissues independent of its ligand *HGF*. Most of the genes that were downregulated in SNU-449 cells by c-Met kinase activity inhibition were elevated in patient derived tumor tissues, significantly. *GYS1*, *H6PD*, *HK2*, *MDH1B*, *PDK4*, *PDPR*, *PFKL*, *PHKG1*, *PKLR* and *SLC2A1 (GLUT1)* gene expressions were significantly higher in tumor tissues than normal tissues (Fig. 5d). Overall survival analysis among Low-*MET* and High-*MET* quartiles showed that patients in the high-*MET* expressing group had significantly shorter survival time (Fig. 5f). Additionally, significant increase of expression in tumor tissues, high expression of *GYS1*, *HK2* and *SLC2A* were significantly correlated with decreased survival of LIHC patients in the TCGA cohort (Fig. 5g).

Furthermore, we performed a comparison analysis between TCGA quartiles that were grouped according to the MET expression levels. We analyzed expression of *HGF*, DEGs (*GYS1, H6PD, HK2, MDH1B, PDK2, PDK4, PDPR, PFKL, PGAM2, PHKG1, PKLR, PYGM* and *SLC2A1*), additional isoforms of hexokinases (*HK1*) and glucose transporters (*SLC2A2, SLC2A3*) which are reported to be expressed in HCC cells and tissues in previous studies^29,30^. *H6PD*, *PDK2, PDK4, PDPR, PKLR* and *SLC2A2* expressions were significantly higher in high-*MET* expressing groups whereas *GYS1, HK1, HK2* and *SLC2A1* expressions were significantly lower. There was not any significant change in the expressions of *HGF, MDH1B, PFKL, PGAM2, PHKG1, PYGM* and *SLC2A4* among quartile groups (Fig. 5e).

In conclusion, c-Met inhibition downregulates expression of genes that take part in fueling the glucose metabolism but, its effect on particular genes is context and cell type dependent (Supplementary Table 1). Tumor tissues with high c-Met expression level in TCGA have increased expression of *H6PD, PDK2, PDK4, PDPR, PKLR, SLC2A2* genes whereas decreased expression of *GYS1, HK1, HK2, SLC2A1*, significantly. Overall, c-Met expression levels and activation status have regulatory effects on glucose metabolism gene expression.

## Discussion

HCC is a complex disease and treatment options remain limited with little progress over the last decades. There is an alarming increase in the incidence of metabolic disorders characterized by elevated levels of blood glucose including diabetes and obesity that reflects the increase in the incidence of HCC^31^.

**Figure 6:**
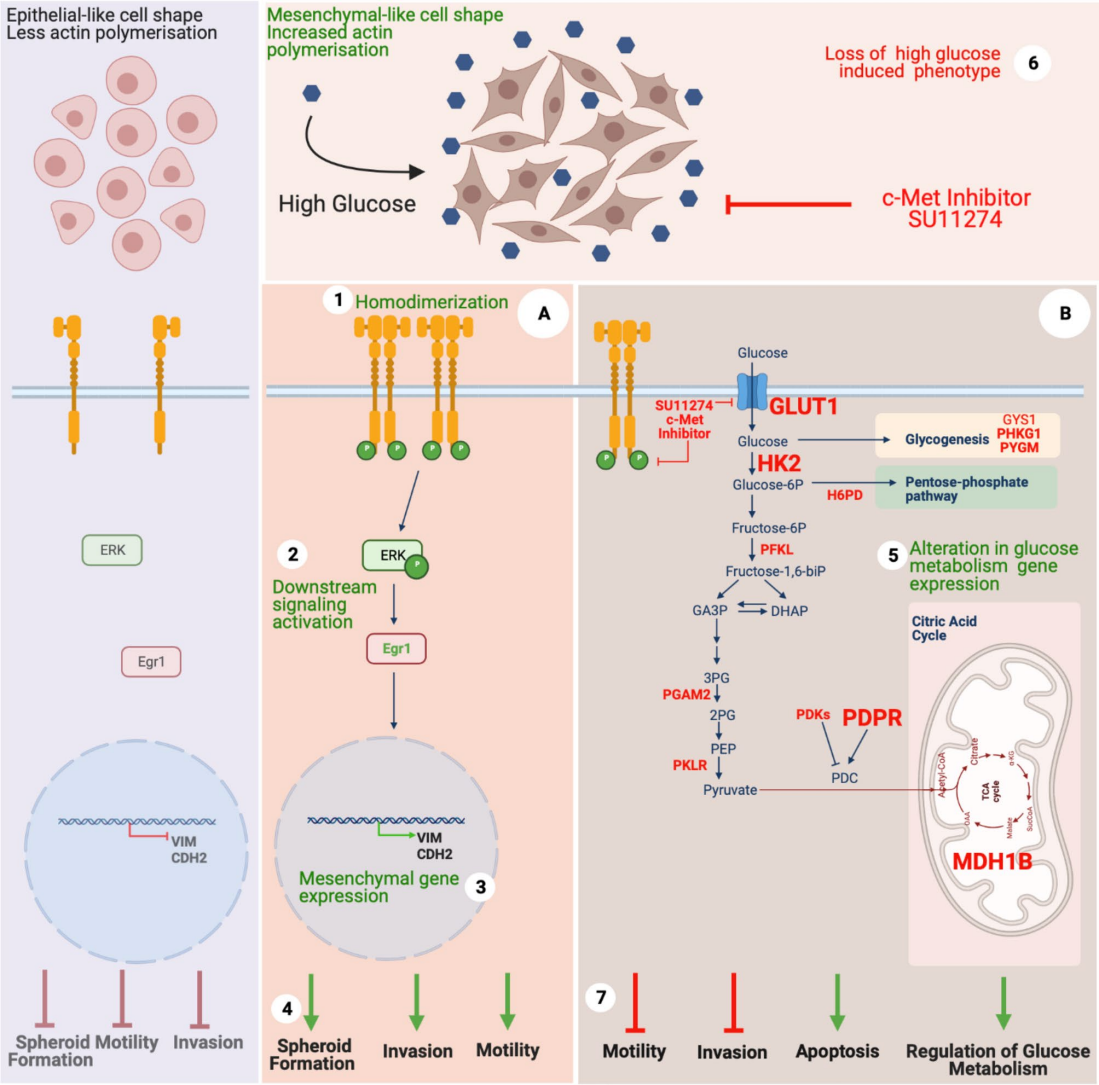
Graphical illustration of high glucose induced EMT via c-Met activation, its downstream signaling and associated biological cellular responses in high glucose and c-Met inhibited conditions. Green and red colored mechanisms represent active and inhibited c-Met signaling conditions, respectively. Molecular mechanisms defined in this study are summarized and numbered as following: (**1**) High glucose induces ligand-independent homodimerization of c-Met which in turn (**2**) activation of c-Met and downstream signaling, (**3**) promotes mesenchymal phenotype via inducing Vimentin (*VIM*) and N-Cadherin (*CDH2*) expression, (**4**) induces motility, invasion and spheroid formation ability. (**5**) Inhibition of c-Met activity by a small molecule inhibitor (SU11274) alters expression of glucose metabolism genes, (**6**) reverses high glucose induced mesenchymal-like phenotype and (**7**) aggressive behavior and eventually sensitizes HCC cells to glucose toxicity and promotes apoptosis. c-Met activated (**A**) and inhibited (**B**) conditions. Glucose metabolism genes which are confirmed with only one cell line are typed in with standard fonts, two cell lines are typed in with “bold” fonts and the genes confirmed in both *in-vitro* and bioinformatic analyses are typed with “big bold” fonts. Created with BioRender.com.

It has been known that consistent high glucose levels in the bloodstream cause glucose toxicity in normal cells leading to cellular damage and organ dysfunction^16^. However, in cancer cells there is a huge demand on energy for rapid proliferation and expansion that are mostly provided by utilization of glucose^32^. Disturbance of blood glucose has been reported to be significantly associated with recurrence and poor survival of HCC patients^6^. Several studies highlight the importance of lowering blood glucose in HCC patients for improving survival, the use of metformin and glucose starvation are among such strategies that were shown to significantly decrease HCC migration, invasion and EMT^33,34^. Therefore, efforts in keeping the blood glucose levels low are expected to contribute to patient survival in many dimensions and have the potential to serve as an Achilles’ heel in cancer therapy. Several studies support the role of increased glucose levels in the activation of EMT and the metastatic phenotype through promoting aberrant regulation of signaling pathways^7–9^. However, the contribution of hyperglycemia to EMT mediated progression in HCC and related mechanisms remains still unclear. Recalling, highly metastatic nature of the HCC, we investigated the link between hyperglycemia and EMT that are critically important in the acquisition of aggressive phenotype, and identified that high glucose treatment: (i) induced ligand independent homodimerization of c-Met (ii) activated Tyr1234/1235 phosphorylation of c-Met and downstream signaling. (iii) promoted EMT, (iv) induced motility and invasion, as well as spheroid formation. (v) Inhibition of c-Met kinase activity altered expression patterns of genes that play roles in glucose metabolism, (vi) reversed high glucose induced aggressive phenotype and (vii) increased apoptotic cell death under high glucose treatment (Fig. 6).

c-Met signaling pathway plays a pivotal role in embryonic development and through life via mediating complex biological processes including cell survival, proliferation, EMT, migration, invasion and morphogenesis^10–12^. Aberrant activation of c-Met signaling contributes to tumor progression and recurrence as well as therapy resistance in HCC via inducing EMT like properties and protecting cells from apoptosis^10,11^. Induction of mesenchymal phenotype and aggressive behavior are well-defined consequences of c-Met activation in HCC^18–20^. We and others identified that c-Met is mostly activated by ligand-independent mechanisms in HCC including chromosomal or mutational re-arrangements, receptor crosstalk, noncoding RNAs, and heparan sulfate proteoglycans^11,35^. In this study, we also determined that c-Met signaling was activated in a glucose (25 mM) rich microenvironment in HCC. Although high glucose (40 mM)- induced c-Met activation has been previously reported in cultured renal epithelium cells^36^, there was not any evidence that explains the potential mechanism behind receptor activation. Indeed, to the best of our knowledge, there is no study evaluating involvement of high glucose treatment in acquisition of aggressive phenotype via regulating c-Met signaling in HCC. Our data demonstrated that high glucose-induced activation of c-Met signaling leads to EMT and changes glucose metabolism gene expression which in turn provides survival advantage to HCC cells. Jin et al. also demonstrated that receptor tyrosine kinases (RTK) including c-Met preferentially rewired the metabolic network and protected BAF3 isogenic cells from glucose deprivation induced cell death^37^. Overall, while high glucose induces cell death in normal epithelial cells, c-Met activation provides survival advantage to cancer cells when increased glucose availability is fluctuating in the environment. Our data also showed that HCC cells developed survival mechanisms via c-Met and were able to survive when glucose is deprived or abundant in the microenvironment. Importantly, cells cultured in high glucose supplemented conditions underwent apoptosis when c-Met kinase activity was inhibited by a specific inhibitor. Another important outcome of c-Met inhibition under high glucose conditions was reversion of EMT and inhibition of aggressive phenotype.

During progression of EMT, cellular metabolism is fine-tuned to meet the increased bioenergetic demands of the cell getting through the challenges of phenotype transition^38,39^. c-Met activation synergistically cooperates with signaling pathways involved in metabolic reprogramming of cancer cells in HCC cells. The cause and consequence link between metabolic reprogramming of the cell and EMT is still an ongoing discussion but accumulating data show that they are interdependent programs^13^. Increased motility of mesenchymal cells generates a significant demand for energy metabolites in mesenchymal cells when compared with their epithelial counterparts. Hyperglycemic microenvironment enhances increased glucose uptake and glycolysis in cancer cells and maintains an acidic microenvironment that selects motile and invasive cells^40–43^. From this point of view, we performed bioinformatic analysis to see whether c-Met has a role in reprogramming of metabolism-related gene expressions in TCGA-LIHC primary HCC tissues. Bioinformatic analysis pointed out that genes (*GYS1, H6PD, HK2, MDH1B, PDK4, PDPR, PFKL, PHKG1, PKLR* and *SLC2A1*) that regulates both glycolysis, glycogenesis and citric acid cycle are elevated in liver tumor tissues compared to normal liver tissues. These findings imply that HCC metabolism is flexible and can operate glycolysis and OXPHOS preferentially to adapt the mechanisms of energy production to microenvironmental changes as well as differences in tumor energy needs. In order to further analyze the regulatory role of c-Met expression level on glucose metabolism, we grouped tumor tissue samples into quartiles according to their *MET* expression levels to compare the four distinctive groups. As expected, there was a significant difference in the expression pattern of genes that plays a role in metabolism and metabolic diseases when “Low-*MET*” expressing and “High-*MET*” expressing HCC tissues compared to each other. Some of these genes (*H6PD*, *PDK2, PDK4, PDPR, PKLR* and *SLC2A2)* were specifically higher in high c-MET expressing groups. Also, we observed a gradual increase into quartiles relative to their *MET* expression level. Despite the fact that, there are some studies reported HGF-induced glycolytic phenotype in head and neck squamous cell carcinoma^44,45^ or hepatocellular carcinoma cells^27^, in accordance with our in vitro findings, HGF expression among the groups was not different.

When we further analyzed the regulatory role of c-Met signaling on glucose metabolism genes via gene expression array and RT-qPCR upon c-Met activity inhibition we have observed that this effect is mostly cell type or context dependent. Among the many genes that we analyzed, expressions of *GYS1, H6PD, HK2, MDH1B, PDK2, PDK4, PDPR, PFKL, PHKG1, PKLR* and *SLC2A1* genes were downregulated by c-Met inhibitor treatment in SNU-449 cell line. Whereas in SK-HEP-1 cells, four of these genes, *HK2, MDH1B, PFKL and SLC2A1, were* downregulated upon c-Met activity inhibition.

These findings further support the fact that HCC metabolism is flexible and c-Met signaling seems to be important to attain this metabolic flexibility depending on the cell type and environmental conditions. Jin et al. reported that labeled glucose was mostly incorporated with lactate and alanine in *MET*-overexpressing BAF3 cells^37^ which supports the potential of c-Met fueling glycolysis and promoting Warburg effect driven metastasis. Additionally, Huang et al. reported ligand dependent c-Met activation promotes Warburg effect by increasing glucose consumption, lactate production and DNA synthesis of HCC cells^27^.

Taken together, the modulatory role of c-Met activity on expression of indicated glucose metabolism genes in HCC is reported for the first time in this study. Together with the literature, there is still more room to investigate the role of c-Met in glucose metabolism related gene expression and cancer cell metabolic regulation in detail with both cancer progression and therapy resistance perspectives. Increasing numbers of data on the effect of c-Met pathway on glucose metabolism of cancer cells is accumulating and our study points out the potential targets for the first time and emphasizes the role of c-Met through a ligand-independent activation mechanism in hyperglycemic conditions. Future studies with animal models and patients are better to be planned to enlighten this critical phenomenon.

## Materials and methods

### Cell culture

All Human HCC cell lines were kindly provided by Prof. Mehmet Öztürk (IBG Center, Izmir, Turkey). SNU-449, SK-HEP-1, HepG2 and HuH-7 cell lines were cultured as described previously^46^. All cell lines were authenticated by STR profiling and tested for mycoplasma infection and confirmed as negative before the experiments. SU11274 (Calbiochem, 448101) was solubilized in DMSO and used with 2.5 µM concentration. HCC cells starved with 2% FBS supplemented culture media without glucose for 24 h before the experiments to down-regulate basal c-Met activity. After starvation, cells were treated with high glucose (25 mM) and 2% FBS supplemented culture media for 16 h. Details of cell culture media and supplements are listed in Supplementary document.

### mRNA isolation

NucleoSpin RNA Isolation kit (Macherey–Nagel, Germany) was used for mRNA isolation according to the manufacturer’s instructions. mRNA was eluted with DNAse/RNAse free distilled water and its concentration was measured by NanoDrop.

### Reverse transcription and RT-qPCR

SensiFast™ cDNA Synthesis Kit (Bioline Meridian Bioscience, USA) was used to transcribe cDNA from mRNA. qPCR reactions were performed with primers (Supplementary Table 2) designed specifically for cDNA sequences of gene of interest (CDS) from NCBI-Genbank. RT-qPCR analysis was performed as described previously^12^ via using SensiFast™ SYBR No-ROX kit (Bioline Meridian Bioscience, USA) with ABI 7500 Fast Real-Time PCR System (Thermo Scientific, USA). At least three biological experiments were performed to analyze and do statistical analysis.

### Immunoblotting

The cells were lysed with RIPA lysis buffer (50 mM Tris–CL pH 7.4, 150 mM NaCI, 1 mM EDTA pH 8.0, 1% NP-40) containing 1 mM Na_3_VO_2_, 1 mM NaF, 1× phosSTOP (Roche Diagnostics, Indianapolis, IN, USA) and 1× protease inhibitor cocktail (Roche Diagnostics, Indianapolis, IN, USA) and the lysates were subjected to immunoblotting assay as described previously^46^. Primary antibodies are listed in Supplementary Table 3. At least three biological replicates per experimental setup were performed for each HCC cell line tested. Immunoblots were developed with LI-COR Odyssey CLx and Biorad ChemiDoc MP imaging systems. Brightness and contrast adjustments of the gel/membrane images were performed with Keynote presentation software in MacOS. Unprocessed images of gels and membranes are presented in Supplementary document.

### Immunofluorescent analysis of the cells

HCC cells were grown on glass cover-slips and after experimental conditions were set, cells were stained with fluorochrome-labeled antibodies as described previously^19^. Imaging was performed with up-right fluorescence microscope (Olympus—BX61) and Zeiss LSM 880-Confocal Laser Scanning Microscopy with Airyscan at IBG Optic Imaging Core Facility. Two biological replicates per condition were analyzed. Corrected total cell fluorescence (CTCF) measurements of Phalloidin staining was performed with biological image analysis program Fiji^47^.

### Trans-well motility and invasion assays

Trans-well inserts with 8 µm pore size (SPL Life Sciences, #37224, Korea) were used to analyze motility and invasion ability of HCC cells. Trans-well motility and invasion experiments were performed as described in previous study^46^. Two biological replicates (each has three or four technical replicates per condition) were conducted to generate statistical analysis.

### Wound-healing assay

Cells were seeded on 24-well tissue culture plates with concentrations sufficient to reach 70%-80% confluency next day. Cells were incubated for 24 h with 2% FBS supplemented culture media without glucose supplementation. Scratching performed with 200 μl pipette tips. Wells were washed with medium twice to remove cells detached with scratching. Fresh 2% FBS supplemented culture media without glucose supplementation was added to the wells and cells were incubated for 24 h. During this period, the gap was imaged in real-time via an automated microscope for live cell imaging (CellDiscoverer7, ZEISS) and images of time-points 6 h, 12 h and 24 h were analyzed for wound closure analysis. Gap distance was calculated via ImageJ program and the results were analyzed by Microsoft Excel. Experiments without real-time imaging equipment were performed as the same and images were collected before and after 24-h incubation. Three biological replicates (each has three or four technical replicates) were conducted to generate statistical analysis.

### Hanging drop spheroid formation assay

HCC cells (2500 cells) cultured in 20 µl of cell culture media supplemented with experimental conditions for 72 h and spheroids were imaged under Zeiss Stemi 508 stereo microscope. Experiment was conducted as previously described^12^. Two biological replicates (each has 40 hanging-drops per condition) were performed to generate statistical analysis.

### Phalloidin staining

HCC cells were seeded on glass cover slips and incubated overnight for adhesion. Alexa Fluor™ 488 Phalloidin (Invitrogen, #A12379 ) staining was performed as previously described for adhesion. Alexa Fluor™ 488 Phalloidin (Invitrogen, #A12379 ) staining was performed as previously described^48^. Imaging was performed at IBG Optic Imaging Core Facility with fluorescent microscope (Olympus—BX61).

### ELISA

After 24 h incubation with starvation media (2% FBS , without glucose), HuH-7, HepG2, SNU-449, SK-HEP-1 cells were cultured for 16 h with culture media supplemented with experimental conditions. Conditioned-media was collected and analyzed with HGF Human ELISA Kit (Life Technologies, #KAC2211) to measure secreted HGF concentration. Three biological replicates (each has five technical replicates) were conducted to generate statistical analysis.

### Crosslinking and immunoprecipitation (IP)

HuH-7, HepG2, SNU-449 and SK-HEP-1 were seeded on 150 mm dishes. After experimental conditions set, cells were washed with 1× PBS for three times and cross-linked by culturing with 0.5 mM ethylene glycol bis (sulfosuccinimidyl succinate) (SULFO-EGS) (Pierce, 21565) for 2 h at 4 °C. c-Met antibody was used to precipitate c-Met protein and protein G Sepharose beads (GE Healthcare, #17_0618_01) were used to capture protein-antibody conjugate. Immunoprecipitation experiments were performed as previously described^18^. Two biological replicates were performed.

### Analysis of gene expressions in TCGA-LIHC dataset

Expression of MET, HGF and glucose metabolism genes in normal and tumor tissues were performed with UALCAN^28^ and Kaplan–Meier survival graphs were generated by GEPIA^49^. Normalized mRNA expression data of HCC tumor tissues in TCGA-LIHC dataset was downloaded from cBioPortal^50,51^ and samples were grouped into quartiles according to the level of normalized *MET* expression counts. Pathway^24^ and disease-gene network (DisGeNET)^25^ enrichment analyses were performed with ClusterProfiler^24^ package in R. Visualization of the fold difference of differentially expressed genes between low-*MET* expressing quartile (Q1) and high-*MET* expressing quartile (Q4) in KEGG pathway was performed with ClusterProfiler package in R. The results reported here are in whole or part based upon data generated by the TCGA Research Network: https://www.cancer.gov/tcga.

### PCR array

RT^2^ Profiler PCR arrays for glucose metabolism genes were provided from Qiagen and analysis was performed according to the manufacturer’s recommendations. mRNA isolation and cDNA synthesis for PCR arrays were performed with miRNeasy RNA isolation kit (Qiagen) and RT^2^ First Strand Kit (Qiagen), respectively. Integrity and quality of isolated mRNA was confirmed with formamide supplemented denaturing agarose gel electrophoresis and confirmed samples were progressed through cDNA synthesis. Three biological replicates were conducted to generate array analysis.

### MTT assay

HCC cells (1000 cells/well) were seeded on 96-well plates and incubated for 24, 48 and 72 h. MTT analysis was performed as described previously^52^. Measured absorbances were analyzed by Microsoft Excel 2016 and plotted by Prism 8. Three biological replicates (each has five technical replicates per condition) were conducted to generate statistical analysis.

### SRB assay

HCC cells (1000 cells/well) were seeded on 96-well plates. TCA solution was added on cells for fixation. Plates were incubated for an hour in the incubator. Plates were air dried and treated with Sulforhodamine B solution, 1% acetic acid and 10 mM Tris, respectively. Measured absorbances (565 nm) were analyzed by Microsoft Excel 2016 and plotted by Prism 8. Three biological replicates (each has five technical replicates per condition) were conducted to generate statistical analysis.

### Basal ECAR and OCR analysis

For basal OCR and ECAR analysis, 10,000 Hepa1-6 cells were seeded on Seahorse XFe96 (Seahorse Bioscience, Agilent) cell culture plates and cultured within the relevant conditions. Three independent experiments were set as including relevant conditions with > 9 technical replicates. 30 min prior to the assay, cells were given XF Base medium (Seahorse Bioscience, Agilent) supplemented with 1 mM sodium pyruvate, 2 mM L-glutamine, and 10 mM glucose (pH 7.4). Basal ECAR and OCR rates were measured for 4 times.

### Statistical analysis

Statistical analyses were performed using the GraphPad Prism version 8.2.1 for MacOS, GraphPad Software, San Diego, California USA, https://www.graphpad.com. Statistical methods included Analysis of variance (ANOVA), Mann–Whitney, Student’s t-test, one sample t test and linear regression tests. Results with *p* < *0.05* were considered as statistically significant. If significant, significance values of the statistical analysis were represented as following: * p ≤ 0.05, ** p ≤ 0.01, *** p ≤ 0.001, **** p ≤ *0.0001.*

## Supplementary Information

Supplementary Information.
