# Supplementary material for: High glucose induced c-Met activation promotes aggressive phenotype and regulates expression of glucose metabolism genes in HCC cells
Source: Sci Rep. 2021 May 31;11:11376. doi: 10.1038/s41598-021-89765-5 (PMC8166976; doi:10.1038/s41598-021-89765-5)

Supplementary File 1

High glucose induced c-Met activation promotes aggressive phenotype and regulates expression of glucose metabolism genes in HCC cells

Hande Topel[1,2], Ezgi Bağırsakçı[1,3], Yeliz Yılmaz[1,2], Ayşim Güneş[1], Gülsün Bağcı[1,3], Dehan Çömez[1,3], Erkan Kahraman[1,2], Peyda Korhan[1], Neşe Atabey[1,*]

Affiliations

[1] Izmir Biomedicine and Genome Center (IBG), Balcova, 35340, Izmir, Turkey

[2] Department of Medical Biology and Genetics, Graduate School of Health Sciences, Dokuz Eylul University, 35340, Balcova, Izmir, Turkey

[3] Department of Molecular Biology and Genetics, Izmir International Biomedicine and Genome Institute, Dokuz Eylul University, 35340, Balcova, Izmir, Turkey

[*] Corresponding author: nese.atabey@ibg.edu.tr, Tel.: +90-232 299 4172

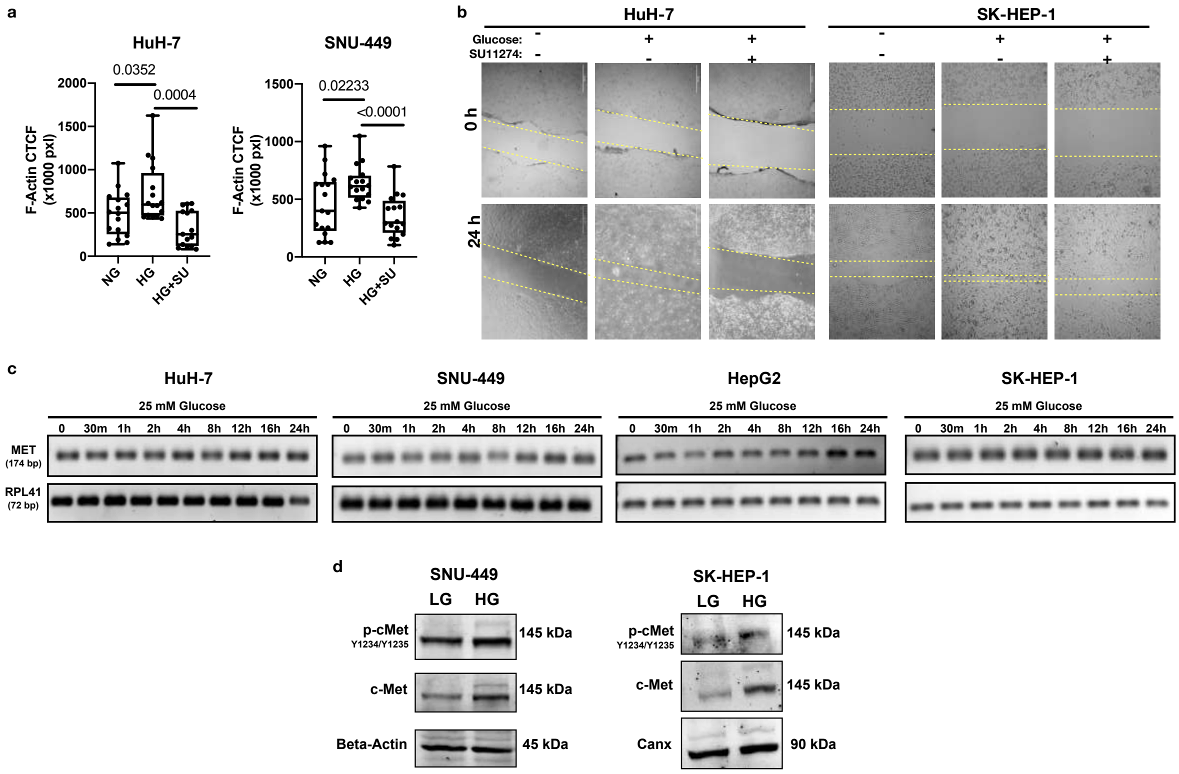

Supplementary Figure 1. Graphical presentation of (**a**) fluorescent intensity measurements for Phalloidin staining. Microscopic image of (**b**) wound closure of HuH-7 and SK-HEP-1 cells after 24-hours incubation in no-, high-glucose and c-Met inhibitor supplemented culture conditions. (**c**) RT-PCR agarose gel electrophoresis image of time-dependent MET and RPL41 expressions in response to high glucose (25 mM) treatment of HuH-7, SNU-449, HepG2 and SK-HEP-1 cells. (**d**) Immunoblotting of c-Met activity phosphorylations (Y1234/Y1235) and c-Met protein in low-glucose (5.5 mM) and high-glucose (25 mM) supplemented conditions of SNU-449 and SK-HEP-1 cells. Boxplot graphs were generated and statistical analyses were performed with the GraphPad Prism version 8.2.1 for MacOS, GraphPad Software, San Diego, California USA, www.graphpad.com. * *$p \leq 0.05$*, ** *$p \leq 0.01$*, *** *$p \leq 0.001$*, **** *$p \leq 0.0001$*

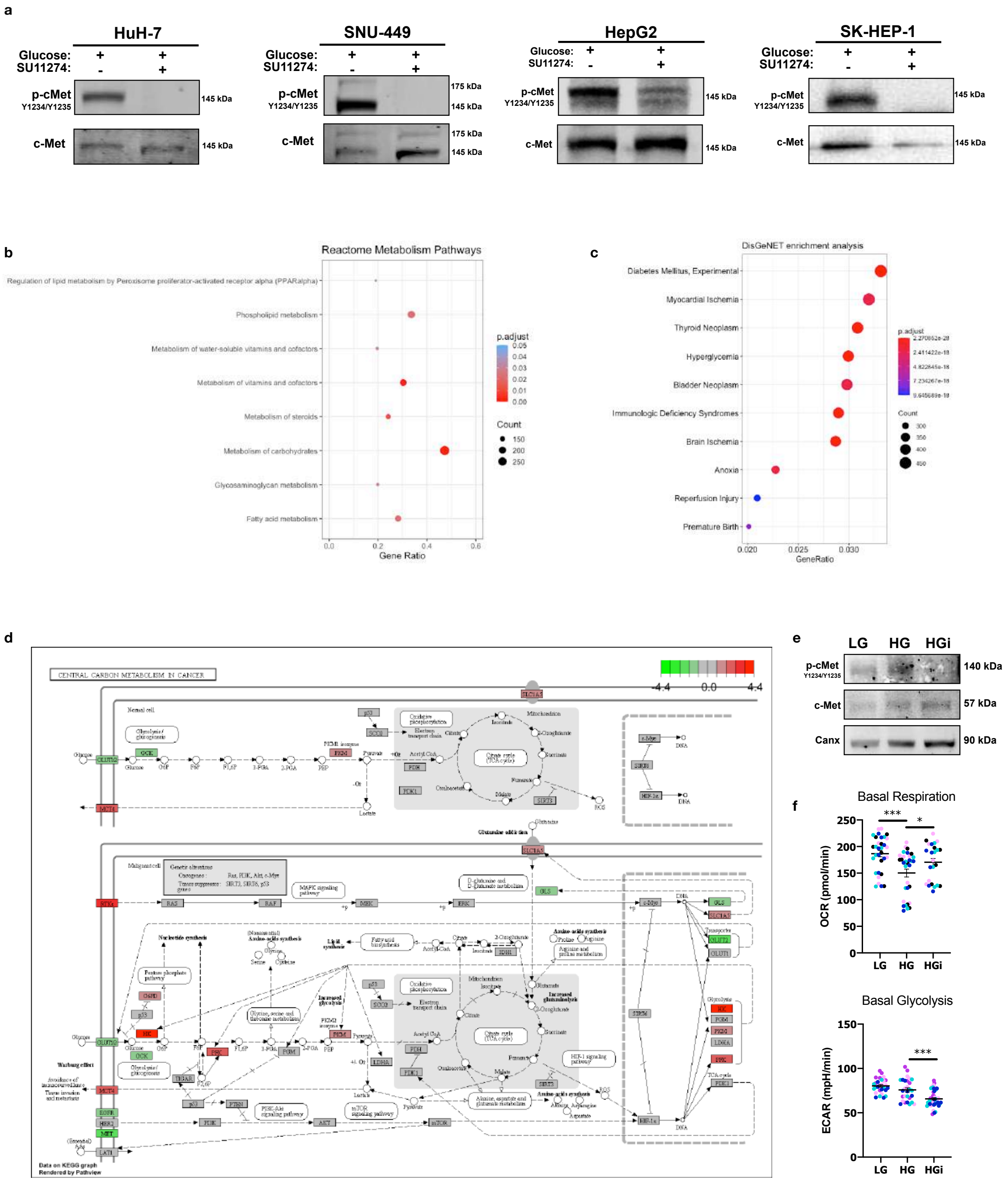

Supplementary Figure 2. (**a**) Immunoblot images of phospho-Met and total c-Met proteins of HuH-7, SNU-449, HepG2 and SK-HEP-1 cells. (**b**) Dot-plot graph of metabolism pathways that were over-represented in pathway enrichment analysis of low-*MET* expressing quartile (Q1) and high-*MET* expression quartile (Q4) of TCGA HCC tissue samples. (**c**) Dot-plot graph of gene-disease association enrichment analysis of low-*MET* expressing quartile (Q1) and high-*MET* expression quartile (Q4) of TCGA HCC tissue samples. (**d**) Visualization of fold

changes of differentially expressed genes in KEGG pathway “central carbon metabolism in cancer”. (**e**) Immunoblot images of phospho-Met, total c-Met and Calnexin proteins of Hepa1-6 cells cultured in low-glucose (LG, 5.5 mM), high-glucose (HG, 25 mM) and high glucose together with c-Met kinase activity inhibitor SU11274 (HGi). (f) Graphs of basal OCR and ECAR levels of Hepa1-6 cells in LG, HG and HGi conditions. Graphs were generated and statistical analyses were performed with the GraphPad Prism version 8.2.1 for MacOS, GraphPad Software, San Diego, California USA, www.graphpad.com. * $p \leq 0.05$, ** $p \leq 0.01$, *** $p \leq 0.001$, **** $p \leq 0.0001$

Supplementary Table 1. Table represents differential expression tendency of gene of interests in RT-qPCR array, RT-qPCR experiments and bioinformatic analyses with TCGA-LIHC dataset. The effect of gene expressions on overall patient survival in TCGA-LIHC dataset is represented in the last (right) column. Glucose metabolism genes that are downregulated in both HCC cell line and upregulated in High-*MET* expressing group of TCGA data are marked with red fonts. Genes that are not investigated in indicated condition/dataset is presented with “hyphen (-)”. “N.S.”: not significant.

GENE	c-Met Activity Inhibition			TCGA-LIHC		
	RT-qPCR Array	SNU-449	SK-HEP-1	Normal vs. HCC Tissues	Low-MET vs. High MET	SURVIVAL
**GYS1**	Downregulated	Downregulated	N.S.	Upregulated	N.S.	Significantly Lower
**H6PD**	Downregulated	Downregulated	Upregulated	Upregulated	Higher in High-MET group	-
**HK1**	-	Downregulated	-	-	Lower in High-MET group	-
**HK2**	Downregulated	Downregulated	Downregulated	Upregulated	Lower in High-MET group	Significantly Lower
**MDH1B**	Downregulated	Downregulated	Downregulated	Upregulated	N.S.	-
**PDK2**	Downregulated	Downregulated	N.S.	N.S.	Higher in High-MET group	-
**PDK4**	Downregulated	Downregulated	N.S.	Upregulated	Higher in High-MET group	-

**PDPR**	Downregulated	Downregulated	N.S.	Upregulated	Higher in High-MET group	-
**PFKL**	Downregulated	Downregulated	Downregulated	Upregulated	N.S.	-
**PGAM2**	Downregulated	Downregulated	-.	Upregulated	N.S.	-
**PHKG1**	Downregulated	Downregulated	N.S.	Upregulated	N.S.	-
**PKLR**	Downregulated	Downregulated	N.S.	Upregulated	Higher in High-MET group	-
**PYGM**	Upregulated	Downregulated	-	N.A.	N.S.	-
**SLC2A1**	Downregulated	Downregulated	Downregulated	Upregulated	Lower in High-MET group	Significantly Lower
**SLC2A2**	-	-	-	-	Higher in High-MET group	-
**SLC2A4**	-	-	-	-	N.S.	-

Cell Culture Media and Supplements:

RPMI 1640 Medium, no glucose, no glutamine (Biological Industries, #01-101-1A)

RPMI 1640 Medium, no glutamine (Biological Industries, #01-104-1A)

DMEM, no glucose, no glutamine (Biological Industries, #01-057-1A)

DMEM, High Glucose (Biological Industries, #01-055-1A)

DMEM, low glucose, no glutamine (Biological Industries, # 01-050-1A)

Glucose Solution (Gibco, #A2494001)

Trypsin EDTA Solution B (0.25%), EDTA (0.05%) (Biological industries, #03-052-1B)

L-Glutamine Solution (200 mM) (Biological industries, #03-020-1B)

MEM Non-Essential Amino Acids Solution (100X) (Biological industries, #01-340-1B)

Sodium Pyruvate (Biological industries, #02-042-1B)

Penicillin-Streptomycin Solution (Biological industries, #01-031-1B)

Fetal Bovine Serum, qualified, heat inactivated (Gibco, #10500064)

Corning® Matrigel® Growth Factor Reduced (GFR) Basement Membrane Matrix, Phenol Red-free, LDEV-free, 10 mL (Corning, #356231)

Supplementary Table 2. Primer sequences used in RT-qPCR analyses.

ZEB1	Forward:	GCACCTGAAGAGGACCAGAG
	Reverse:	TGCATCTGGTGTTCCATTTT
ZEB2	Forward:	CGGTGCAAGAGGCGCAAACA
	Reverse:	GGAGGACTCATGGTTGGGCA
HK2	Forward:	GCTGTGGTGGACAGGATACG
	Reverse:	ATTTCGGAGCCAGGTCCTTC
VIM	Forward:	CCTTGACATTGAGATTGCCACCTA
	Reverse:	TCATCGTGATGCTGAGAAGTTTCG

PFKL	Forward:	TCCTCATCTACGAGGGCTATG
	Reverse:	CGAGCGCTGCCAATGATAGT
PDPR	Forward:	ATCTCCCCCAAGAAAGTGGC
	Reverse:	CATGAAGAACAGATGTCCGGT
MDH1B	Forward:	CGGGTAGAGCAGATTGTCCATA
	Reverse:	CGATCCAACAGCTCTCTCCAG
RPL41	Forward:	GAAACCTCTGCGCCATGA
	Reverse:	TCTTTCTTCTTTTGCGCTTCA
GAPDH	Forward:	GAAGGTGAAGGCGGAGTC
	Reverse:	GAAGATGGTGATGGGATTTC
PKLR	Forward:	CAACTGACCCAGGAGCTG
	Reverse:	CTCCACGGAGCGAGATGC
GYS1	Forward:	ATACTGCATGGAAAGGGCGG
	Reverse:	ACTCCTGGATTCGAGCCTTG
PHKG1	Forward:	ACTGAAGATCTGAGCATGACCC
	Reverse:	ATCGCCTGACCACACTGCTAA
PDK2	Forward:	GACATGGCTAAGCTCCTGTGT
	Reverse:	CGCCCTCATGGCATTCTTGA
PDK4	Forward:	CCAAGCCACATTGGAAGCAT
	Reverse:	CGATGTGAATTGGTTGGTCTGG
H6PD	Forward:	TGCCTTTCCGAGACCAGAAC
	Reverse:	GCGAATGACACCGTACTCCT
SLC2A1 (GLUT1)	Forward:	GCGGGTTGTGCCATACTCAT
	Reverse:	ACCCACTTCAAAGAAGGCCA
CDH-2 (N-Cadherin)	Forward:	ATTGGACCATCACTGGGCTTA
	Reverse:	CACACTGGCAAACCTTCACG
MET	Forward:	GTCAGCCTTGTCCCTCCTTC
	Reverse:	GTCCTGCAGTCAATGCCTCT

Supplementary Table 3. Antibodies used in IF and WB experiments

Cell Signaling	9272	Akt
Cell Signaling	4060	Phospho-Akt (Ser-473)
Santa Cruz Biotechnologies	Sc-11397	Calnexin
Cell Signaling	9101	Phospho-Erk 1/2 p44/42 (MAPK) (Thr202/Tyr204)
Cell Signaling	4377	Phospho-Erk 1/2 p44/42 (MAPK) (Thr202/Tyr204)
Cell Signaling	4696	Erk 1/2 p44/42
Cell Signaling	3127	Met
Santa Cruz Biotechnologies	Sc-161	Met
Cell Signaling	3129	Phospho-Met (Y-1234/1235)
Santa Cruz Biotechnology	Sc-101033	Egr-1
Invitrogen	A-11005	Alexa594-conjugated goat anti-mouse secondary antibody
Invitrogen	A-11037	Alexa594-conjugated goat anti-rabbit secondary antibody
LI-COR Biosciences	926-68071	IRDye 680RD Goat anti-Rabbit IgG Secondary Antibody

LI-COR Biosciences	926-32210	IRDye® 800CW Goat anti-Mouse IgG Secondary Antibody

PDF files of supplementary figures and raw membrane images of immunoblots are presented in the following pages of this document.

Supplementary Figure 1

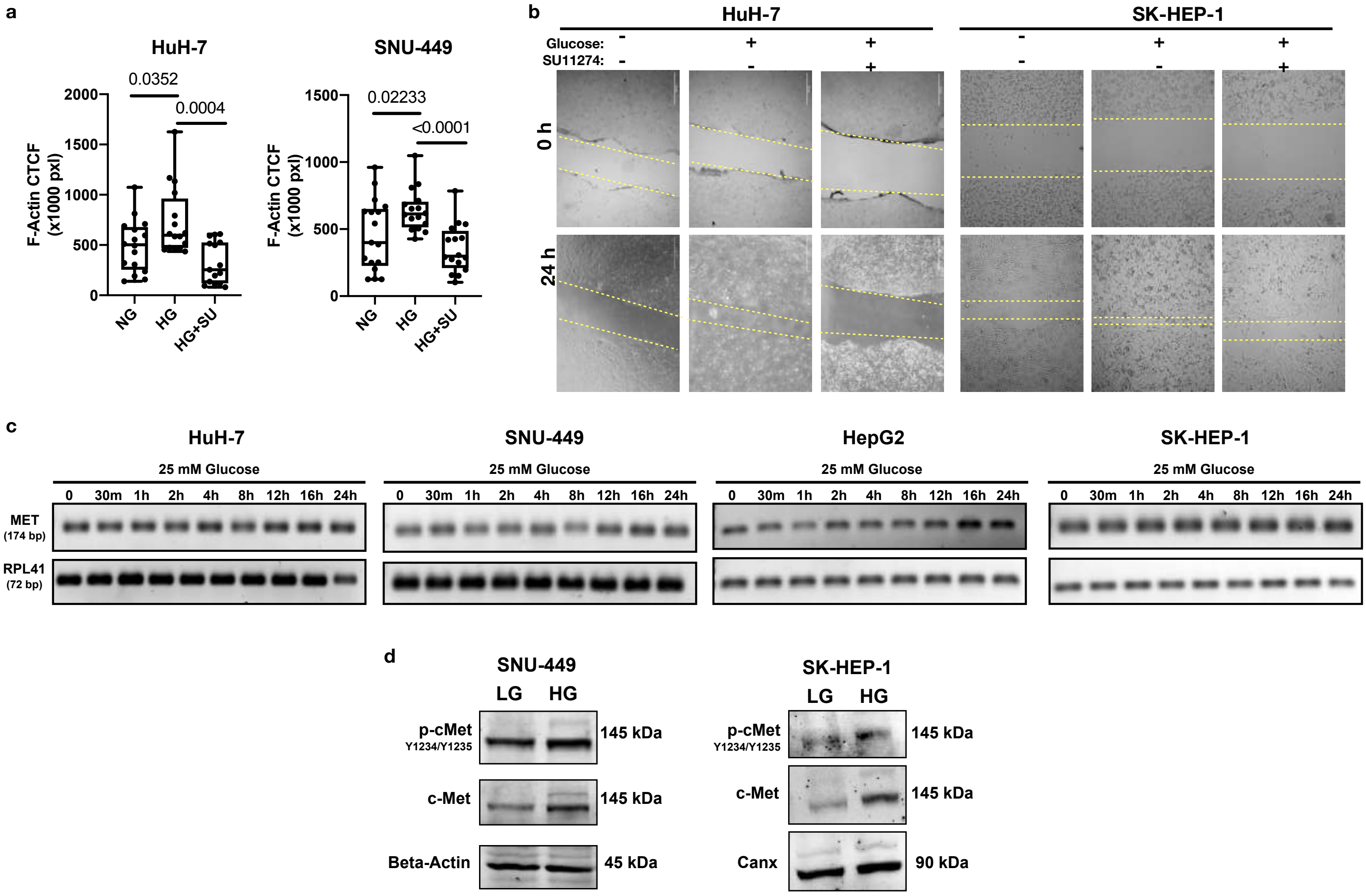

Supplementary Figure 2

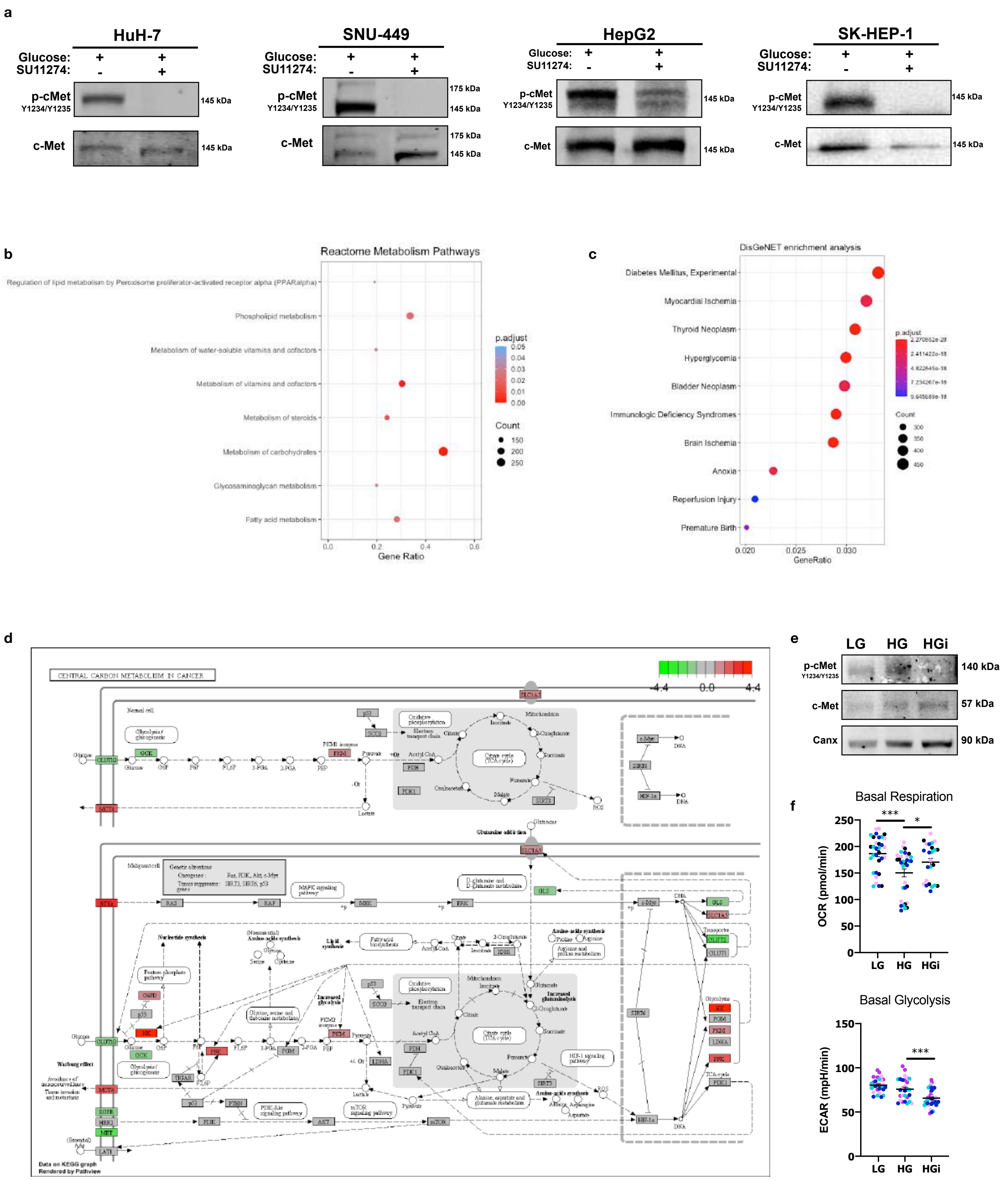

Figure 1 c-d-e

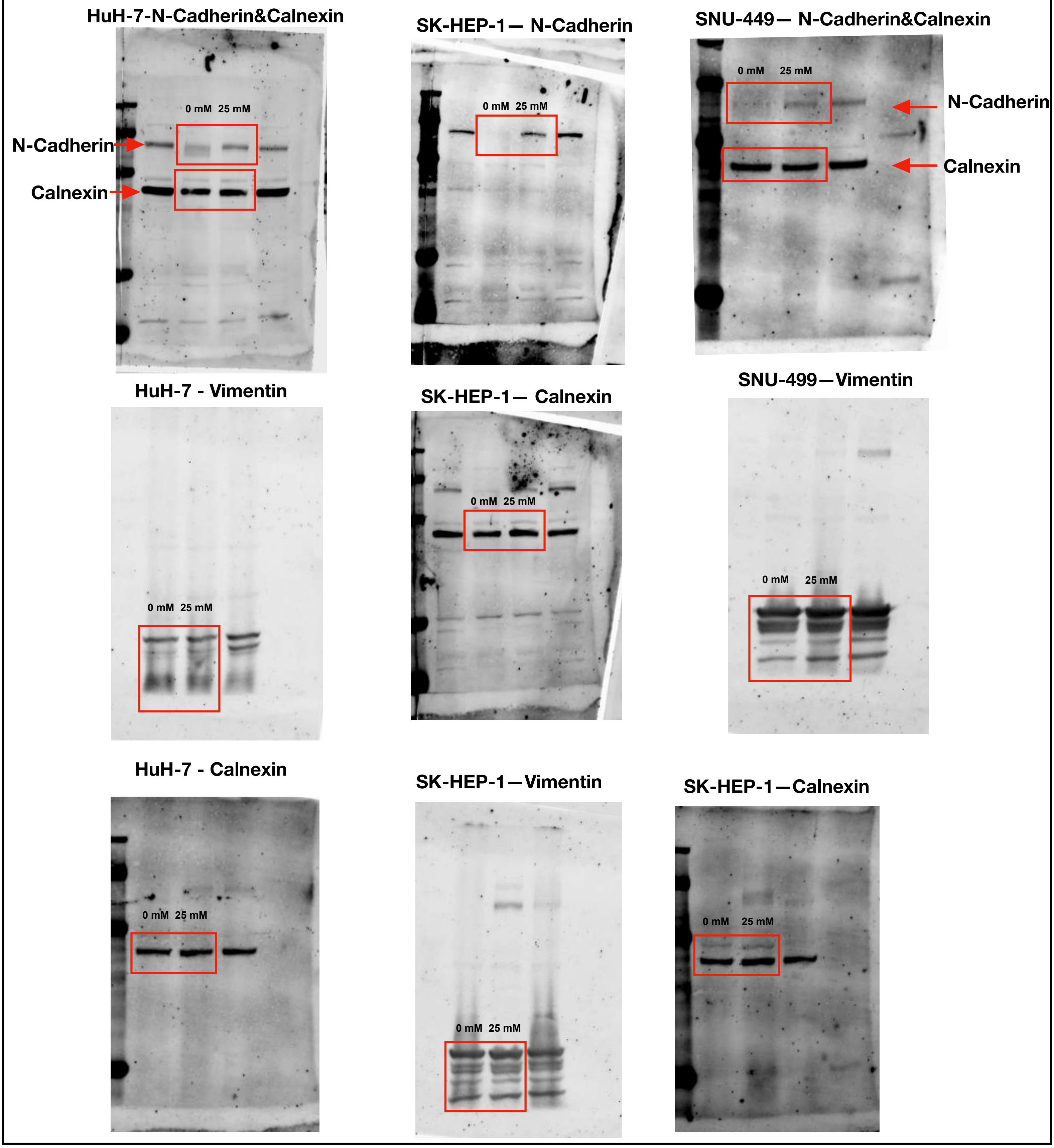
HuH-7-N-Cadherin&Calnexin
0 mM 25 mM
N-Cadherin
Calnexin
SK-HEP-1— N-Cadherin
0 mM 25 mM
SNU-449— N-Cadherin&Calnexin
0 mM 25 mM
N-Cadherin
Calnexin
HuH-7 - Vimentin
0 mM 25 mM
SK-HEP-1— Calnexin
0 mM 25 mM
SNU-499—Vimentin
0 mM 25 mM
HuH-7 - Calnexin
0 mM 25 mM
SK-HEP-1—Vimentin
0 mM 25 mM
SK-HEP-1—Calnexin
0 mM 25 mM

**Membranes are cut according to the molecular marker after transfer and incubated with antibody of interest.*

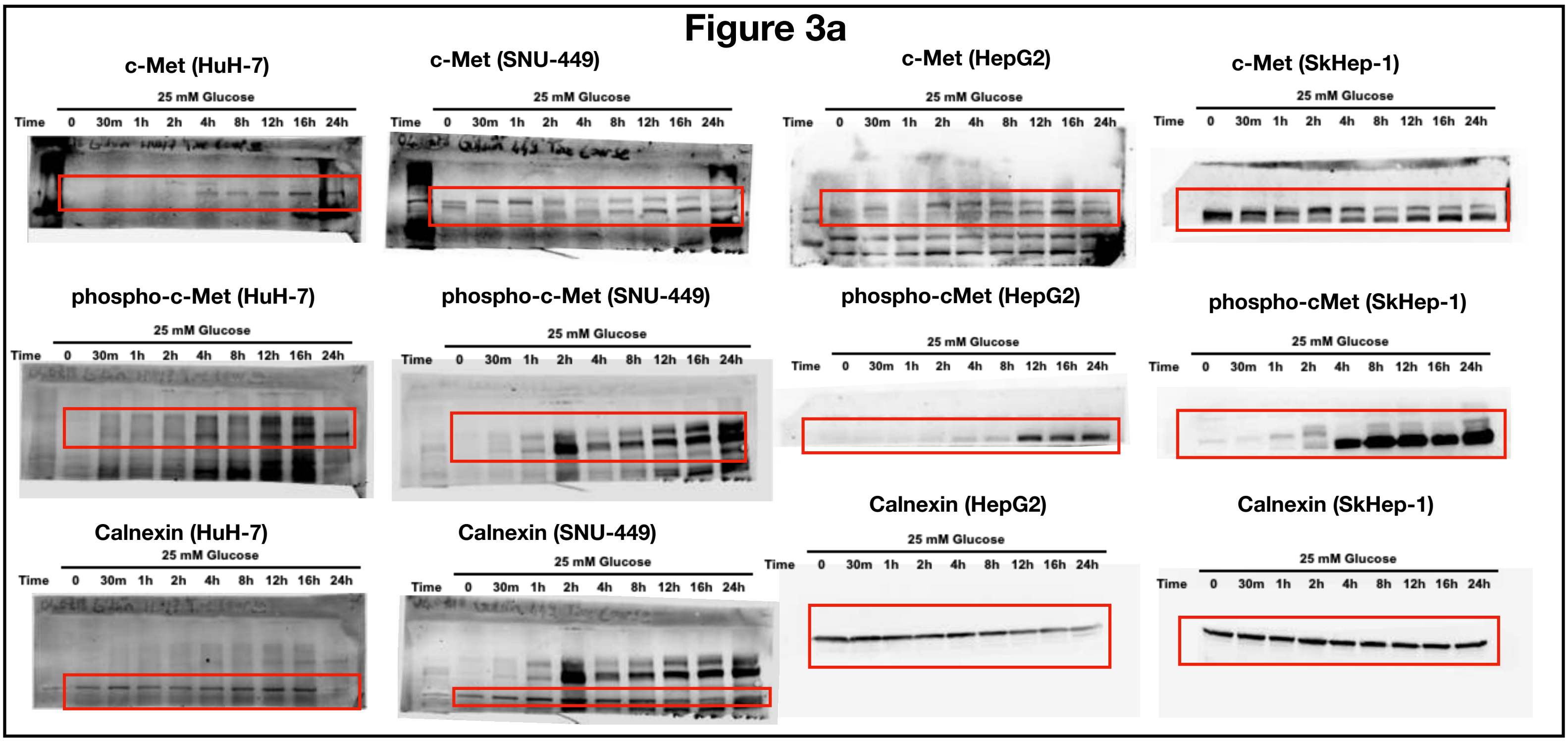

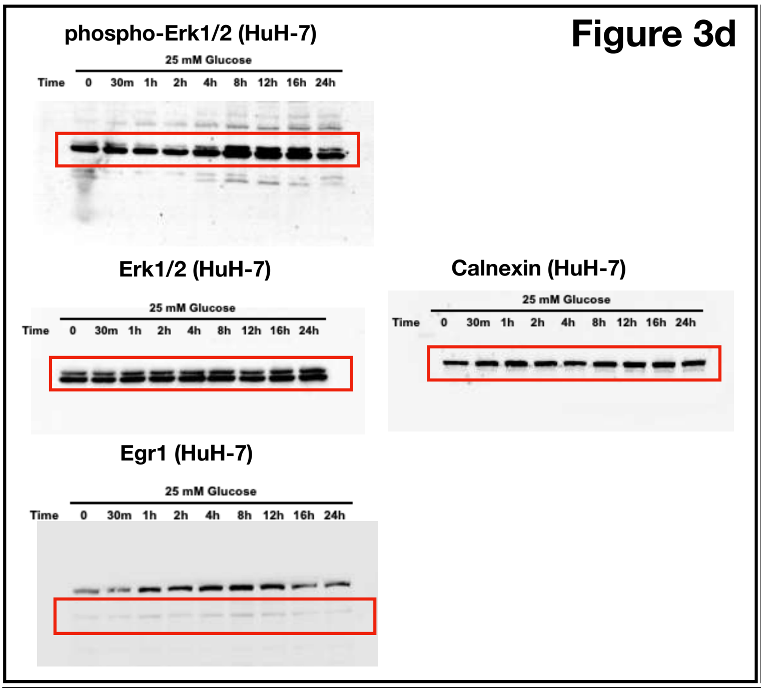

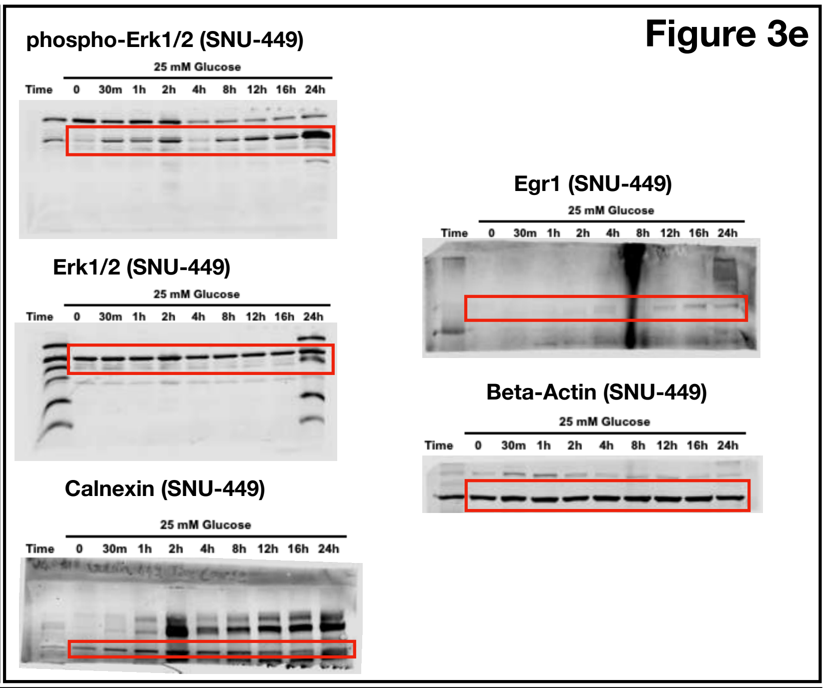

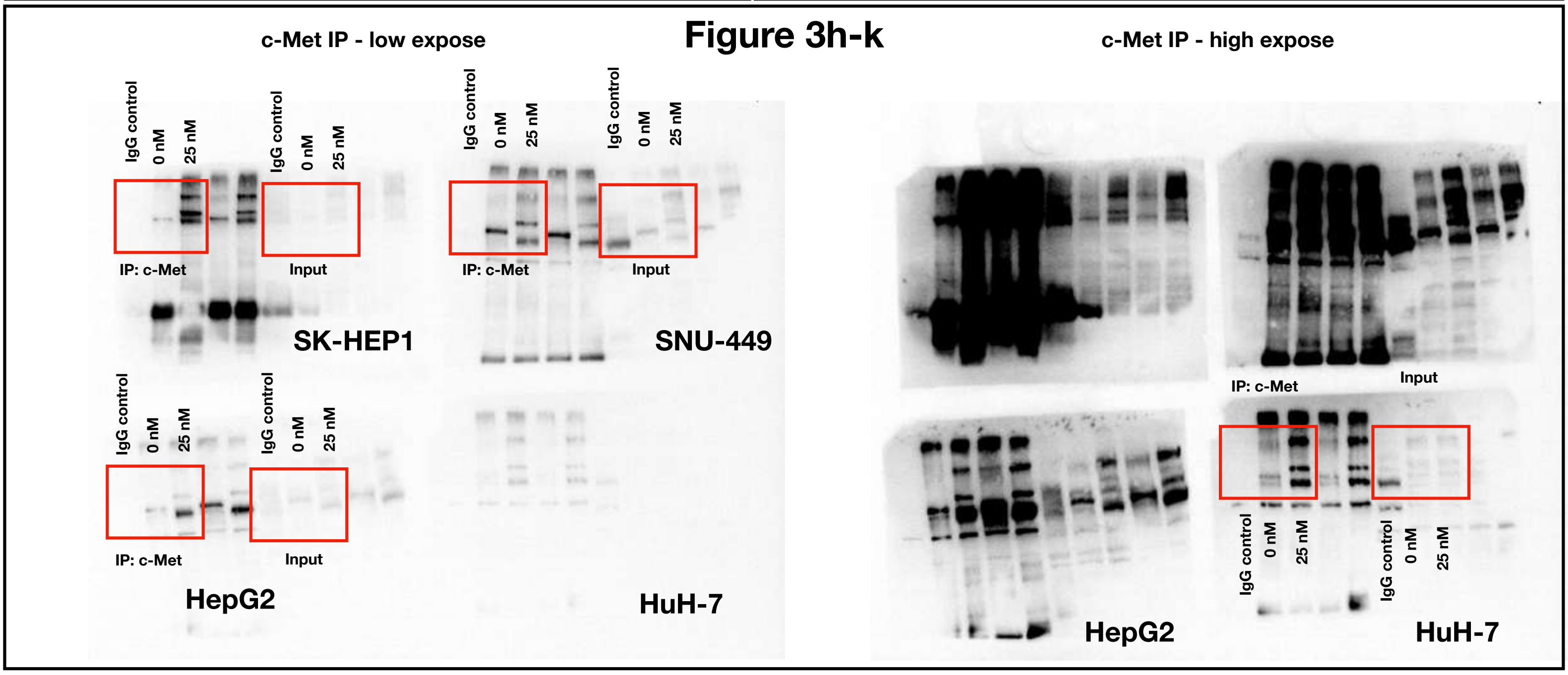

Membranes are cut according to the molecular marker after transfer and incubated with antibody of interest.

Supplementary Figure 1c (agarose gel images)

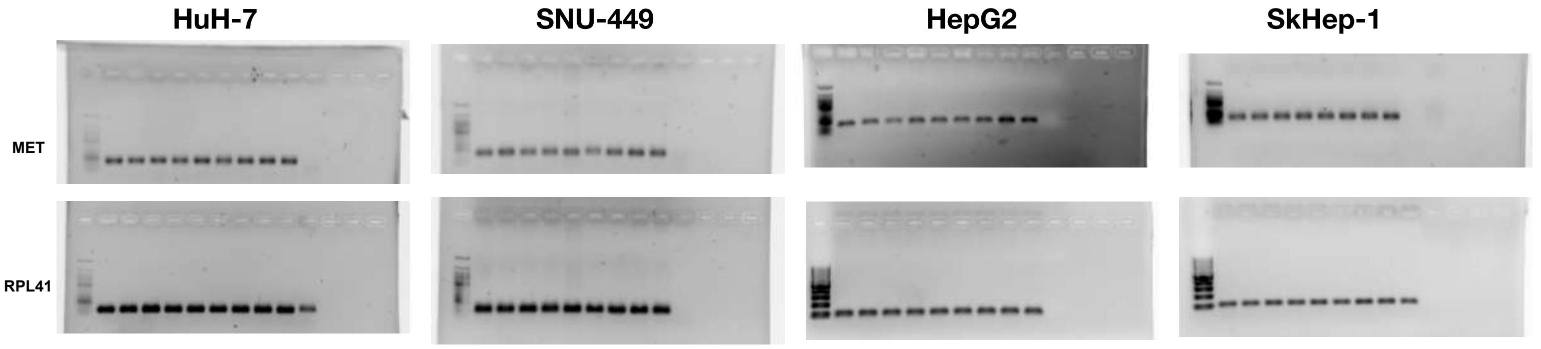

Supplementary Figure 1d

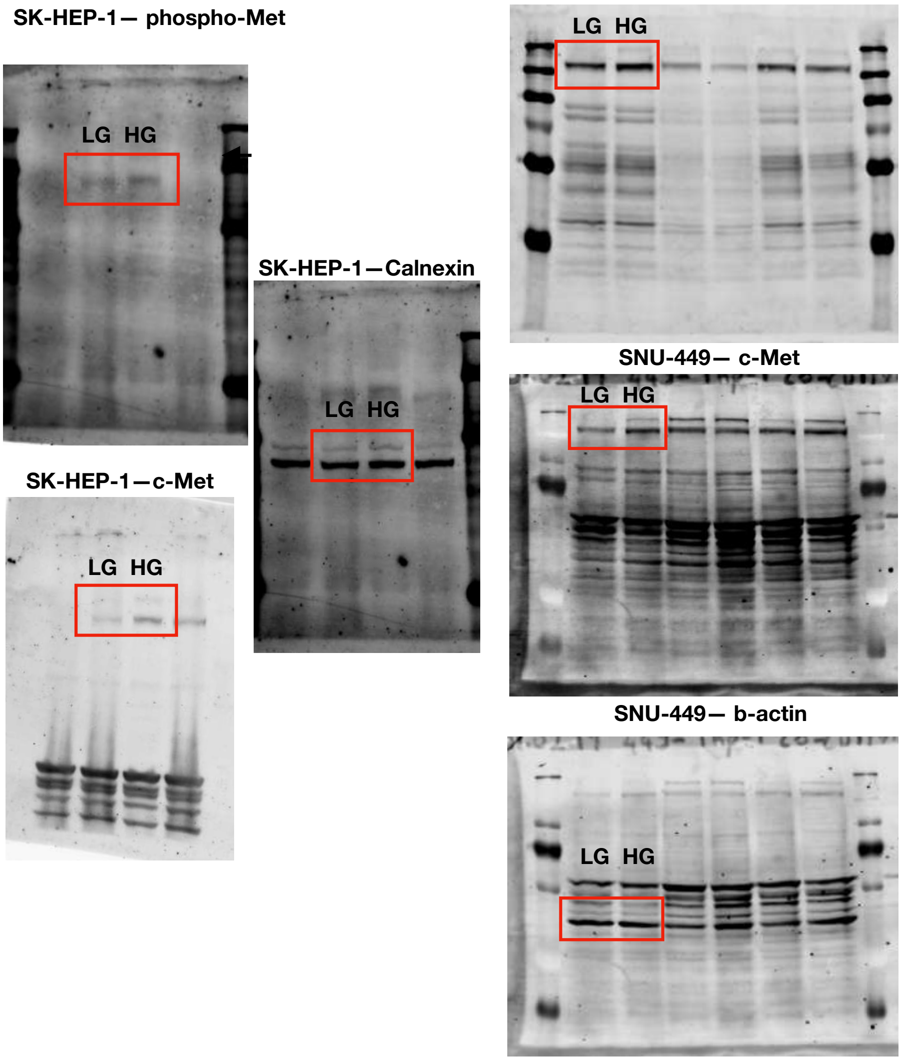

Supplementary Figure 2a

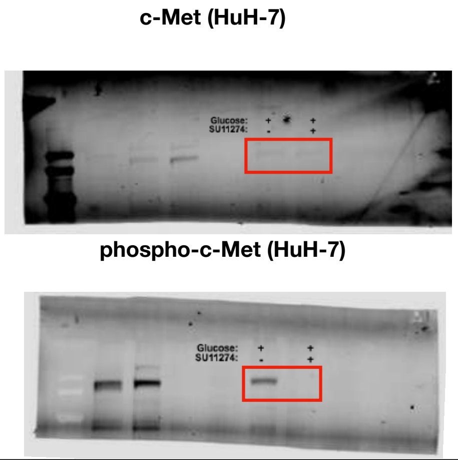

Supplementary Figure 2a

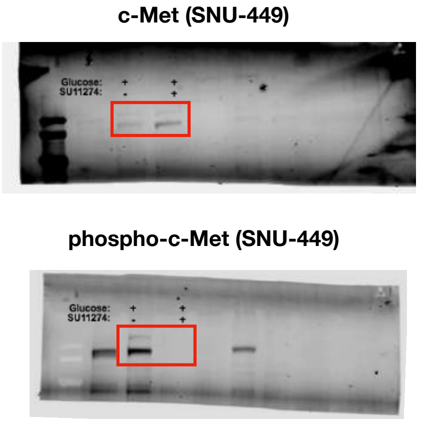

Supplementary Figure 2a

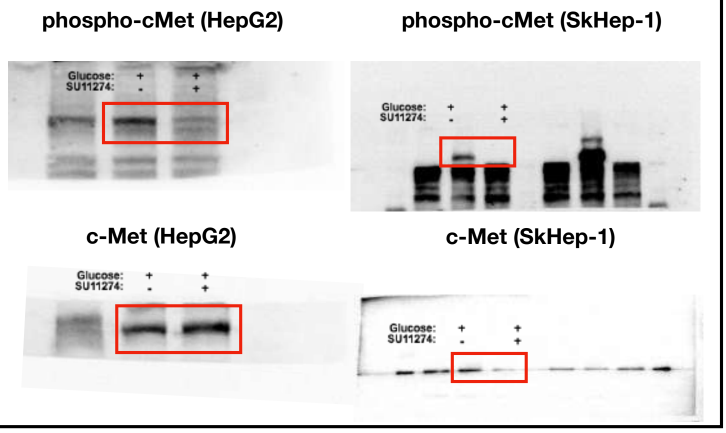

Supplementary Figure 2e

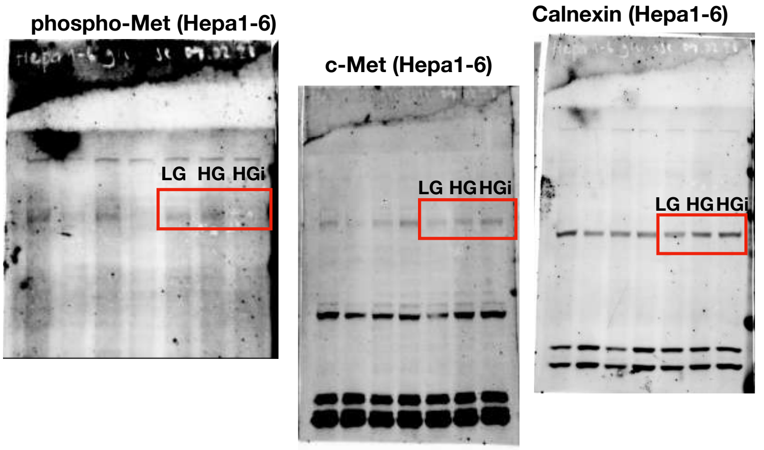

Supplement: Supplementary file 1 — Supplementary Information. [file 41598_2021_89765_MOESM1_ESM.pdf]
